# Advances in Biomarker-Guided Therapy for Pediatric- and Adult-Onset Neuroinflammatory Disorders: Targeting Chemokines/Cytokines

**DOI:** 10.3389/fimmu.2018.00557

**Published:** 2018-04-04

**Authors:** Michael R. Pranzatelli

**Affiliations:** ^1^National Pediatric Neuroinflammation Organization, Inc., Orlando, FL, United States; ^2^College of Medicine, University of Central Florida, Orlando, FL, United States

**Keywords:** acute disseminated encephalomyelitis, multiple sclerosis, neuromyelitis optica, neuropsychiatric lupus, *N*-methyl-d-aspartate receptor encephalitis, opsoclonus–myoclonus syndrome, pediatric neuroinflammatory disorders, Rasmussen encephalitis

## Abstract

The concept and recognized components of “neuroinflammation” are expanding at the intersection of neurobiology and immunobiology. Chemokines (CKs), no longer merely necessary for immune cell trafficking and positioning, have multiple physiologic, developmental, and modulatory functionalities in the central nervous system (CNS) through neuron–glia interactions and other mechanisms affecting neurotransmission. They issue the “help me” cry of neurons and astrocytes in response to CNS injury, engaging invading lymphoid cells (T cells and B cells) and myeloid cells (dendritic cells, monocytes, and neutrophils) (adaptive immunity), as well as microglia and macrophages (innate immunity), in a cascade of events, some beneficial (reparative), others destructive (excitotoxic). Human cerebrospinal fluid (CSF) studies have been instrumental in revealing soluble immunobiomarkers involved in immune dysregulation, their dichotomous effects, and the cells—often subtype specific—that produce them. CKs/cytokines continue to be attractive targets for the pharmaceutical industry with varying therapeutic success. This review summarizes the developing armamentarium, complexities of not compromising surveillance/physiologic functions, and insights on applicable strategies for neuroinflammatory disorders. The main approach has been using a designer monoclonal antibody to bind directly to the chemo/cytokine. Another approach is soluble receptors to bind the chemo/cytokine molecule (receptor ligand). Recombinant fusion proteins combine a key component of the receptor with IgG1. An additional approach is small molecule antagonists (protein therapeutics, binding proteins, and protein antagonists). CK neutralizing molecules (“neutraligands”) that are not receptor antagonists, high-affinity neuroligands (“decoy molecules”), as well as neutralizing “nanobodies” (single-domain camelid antibody fragment) are being developed. Simultaneous, more precise targeting of more than one cytokine is possible using bispecific agents (fusion antibodies). It is also possible to inhibit part of a signaling cascade to spare protective cytokine effects. “Fusokines” (fusion of two cytokines or a cytokine and CK) allow greater synergistic bioactivity than individual cytokines. Another promising approach is experimental targeting of the NLRP3 inflammasome, amply expressed in the CNS and a key contributor to neuroinflammation. Serendipitous discovery is not to be discounted. Filling in knowledge gaps between pediatric- and adult-onset neuroinflammation by systematic collection of CSF data on CKs/cytokines in temporal and clinical contexts and incorporating immunobiomarkers in clinical trials is a challenge hereby set forth for clinicians and researchers.

## Introduction

The era of “biomarker-guided therapy” has introduced a paradigm shift in immunotherapeutics based on the biological make-up of the individual patient (1). Integral to the concept of *personalized medicine* or “precision medicine” is the use of molecular biomarkers to improve detection of neuroinflammation and predict drug benefit or adverse effects (2). Using the approach of a translation paradigm, it affords a rational alternative to traditional trial-and-error, broad-spectrum immunotherapy with its variable successes and failures. Patients with neuroinflammation are extremely heterogeneous, more than clinical phenotypes suggest, and non-targeted interventions using agents that “should work” for the disease, often miss their mark. Other clinical challenges include access and use of more effective methods to diagnose immune dysregulation and neuroinflammation (3), recognizing sustained subclinical disease activity that can foster disease progression (4), and understanding and predicting or preventing relapse. The biomarker-guided approach helps advance preclinical studies to clinical proof-of-concept studies in targeted drug development (2).

Because of cerebrospinal fluid (CSF) proximity to the inflammatory process in brain parenchyma, CSF biomarkers are highly sought in a panoply of disorders with primary or secondary neuroinflammation (3), and those, not abundantly researched blood markers, are not covered here to delimit the scope. Immune cell-specific markers measured through immunophenotyping have greatly aided detection of the cellular “players” in intrathecal inflammation (5), but inflammatory mediators are also a major part of the dynamics in directing the cell to action and orchestrating immunologic contexts (6). *Chemokines* (CKs) and other *cytokines* comprise the major inflammatory mediators, and many are detectable in CSF and qualify as biomarkers that delineate the inflammatory process (7). Breakthrough technological advances have led to the discovery of new molecular entities and made it easier for research labs to measure not just one, but panels of inflammatory mediators simultaneously in a single sample (3, 8, 9). Systematic measurement of CKs and non-CK cytokines would foster the goals of early and maintained targeting of inflammatory mediators and biomarker-guided initiation and monitoring of a drug.

This article is a conspectus on targeting CKs and other cytokines and their receptors or administering them therapeutically. The burgeoning field is so enormous, the review cannot be all inclusive, nor can it keep up with the daily publications and treatment updates. Instead, it purveys selected information considered necessary to consider targeted therapies in paradigmatic diseases and interpret neuroinflammation’s mosaic of clinical facades. Some biomarker-guided immunotherapies for unrelated human disorders are interweaved historically with those used for neuroinflammation or hold such potential for future applications to neuroinflammatory disorders, so they are covered here selectively. Certain other areas are only lightly touched upon or not covered. Extensive information on chemo/cytokines and signal transduction pathways already has been comprehensively reviewed elsewhere (6, 7, 10–14). Neurodegenerative disorders in adults, most of which do not overlap with children, were considered outside the scope. The field of psychoneuroimmunology is rapidly developing, but the article focuses primarily on neuroimmunologic conditions and neuroimmune pharmacology. A highlight of the review stems from the statistic that global central nervous system (CNS) biomarker markets were estimated to increase to $3 billion by 2015, yet most of the biomarker research has been on adult-onset neuroinflammatory disorders, not necessarily providing insight into the disorders that afflict children. Therefore, studies on both pediatric- and adult-onset neuroinflammatory disorders are included herein, with the hope of achieving a balanced view.

The layout of the review reflects its neuroimmune pharmacology orientation. “Cks/Cytokines and Their Receptors” provides a summary of the science behind inflammatory mediator targets; “Comparison of csf ck/Cytokine Immunomarker Profiles in Human Neuroinflammatory Disorders” presents CSF data on target measurements in human disease; “Targeting CKs or Other Cytokines” describes targeted clinical trial experience and ongoing trials; “Considerations in Designing Future Clinical Trials for Neuroimmunologic Application” discusses strategizing about next steps and future goals.

## CKs/Cytokines and Their Receptors

### Non-CK Cytokines

Cytokines comprise 300 soluble low-molecular-weight proteins or glycoproteins, such as interleukin (IL), interferon (IFN), tumor necrosis factor (TNF), colony-stimulating factors (G-CSF, GM-CSF), and other growth factors [tumor growth factor (TGF)] (6, 15). They perform various functions in the immune system in both health and disease. Non-CK cytokines are structurally and functionally diverse, with the largest group having a monomeric alpha structure (IL-2 and IL-4) and the smallest group, a beta structure (IL-1 and IL-18); others may be heterodimeric (IL-12, IL-23, and IL-23). They regulate lymphocytes (IL-2, IL-4, IL-10, and TGF-β), natural immunity (TNF-α, IL-1β, IFN-β, IL-5, IL-10, IL-12, and type 1 INF), and activate inflammatory cells [interferon-gamma (IFN-γ), TNF-α, TNF-β, IL-5, IL-10, and IL-12] (6).

Listing of “pro-inflammatory” vs “anti-inflammatory” is useful but not entirely straightforward, given that cytokine effects may differ depending on the microenvironment. Pro-inflammatory cytokines typically include TNF-α, IFN-γ, IL-1β, IL-2, IL-6, IL-8, IL-12, IL-17, IL-18, IL-23, and IL-36 (16–18). However, TNF-α can also function physiologically at brain synapses (7). Anti-inflammatory/immunoregulatory cytokines encompass IL-4, IL-5, IL-10, IL-13, IL-35, and IL-37 (19, 20).

The TNF cytokine superfamily is one of the largest family of cytokines, consisting of 19 cytokines and almost 30 receptors (21). The cytokines include TNF-α, TNF-β, OX40L, toll-like factor (TL1A), GITRL, TWEAK, RANK1, lymphotoxin (LT-α and LT-β), and many others. B cell-activating factor (BAFF), also called TNF ligand 13B, and a proliferation-induced ligand (APRIL) are members of the TNF family (22).

The IL-1 family includes inflammatory cytokines (IL-1α, IL-1β, IL-18, IL-33, and IL-36), an anti-inflammatory cytokine (IL-37), and receptor antagonists (IL-1Rα, IL-36Rα, and IL-38) (19). The IL-10 family also includes IL-22 (23). The IL-12 family comprises IL-12, IL-23, IL-27, and the anti-inflammatory cytokine IL-35 (20). IL-8 is also CXCL8 and belongs to a CK family (24). The IL-17 superfamily comprises IL-17A to IL-17F, and IL-17 plays a role in autoimmune diseases.

The cytokine network exhibits intriguing properties. It enables chemotaxis through formation of concentration gradients, has *pleotropic effects* (pleiotrophy) and has redundant effects (such as two different cytokines activating a particular cell type) (8). They may be synergistic or antagonistic. Some cytokines affect B cell differentiation and proliferation (IL-2, IL-4, IL-5, IL-6, IL-10 TGF-β, and IFN-γ). Others trigger activation of effector cells, such as CD8+ cytotoxic T cells (IL-2, IL-6, and IL-12) and natural killer (NK) cells (IL-2 and IL-12). Most cytokines act in the local milieu and are short lived. By contrast, M-CSF and TGF-β are able to act at a distance. IL-1α and IL-33 are preformed and released in the presence of tissue injury, therefore acting as *alarmins*.

There are three classes of IFNs: type 1 IFN (α and β), type 2 IFN-γ, and type 3 IFNs. Besides their role in antimicrobial defenses, IFNs activate macrophages and NK cells, an immune function.

The cellular source of non-CK cytokines provides a functional grouping. Cytokines of adaptive immunity are secreted predominantly by various T cells (Th1, Th2, and NKT cells), but also NK cells. Cytokines of innate immunity are produced primarily by macrophages, but also by dendritic cells (DCs), some T cells, and endothelial cells (25). Inflammasome activation is necessary for production of IL-1β and IL-18 (26). Because of the multiplicity of cellular sources of cytokines, the terms *monokines* (monocyte/macrophage-derived) and *lymphokines* (lymphocyte-derived) are less dichotomizing.

#### Cytokine Receptors (CRs)

*Cytokine receptors*, which are divided into several families, reside on target cells and mediate cytokine actions (26). Of these, the cytokine R type 1 and type 2 receptor classes bind the most non-CK cytokines (27). Type 1 CRs are typified by IL receptors; type 2, by IFN receptors. Type 1 and type 2 CRs are distinguished by unusual properties. One is the existence of soluble and *decoy receptors*, which bind cytokines without triggering signaling. Another is production of endogenous selective antagonist molecules, denoted as IL-1Ra, IL-31Ra, and IL-36Ra, which also bind to CRs and prevent signaling. There are also CR subfamilies: IL-12R is a subfamily of the IL-6R family (Table 1) (26).

**Table 1:** Cytokine receptors (CR) and endogenous agonists and antagonists.

Receptor family	Agonists	Antagonists	CR type
**IL-1 family**			
IL-1R1	IL-1α, IL-1β	IL-1Ra	Type 2
IL-1R2			
IL-18	IL-18, IL-37		
IL-33R	IL-33		
IL-36R	IL-36α, IL-36β, IL-36γ	IL-36Ra	

**IL-2 family**			
IL-2R	IL-2	IL-1Ra	Type 1
IL-4R1	IL-4		
IL-4R2	IL-13, IL-4		
IL-7R	IL-7		
IL-9R	IL-2	IL-1Ra	
IL-13Rα2			
IL-15R	IL-15		
IL-21R	IL-21		

**IL-3 family**			
IL-3R	IL-3		Type 1
IL-5R	IL-5		
GM-CSFR	G-CSF, GM-CSF		
IL-3α	IL-3		
IL-5Rα	GM-CSF		
GM-CSFRα	GM-CSF		

**IL-6 family**			
IL-6R	IL-6	Type 1	
IL-11R	IL-11		
IL-27R	IL-27		
IL-31R	IL-31		
IL-6Rα	IL-6		

**IL-10 family**			
IL-10R	IL-10		Type 2
IL-20R	IL-19		
IL-22Rα/20β	IL-20		
IL-22Rα/10β	IL-22		
IFN-λ	IFN-λ1, IFN-λ2, IFNλ3		

**IL-12 family**			
IL-12R	IL-12	A/IL-12B	Type 1
IL-23R	IL-12B/IL-23		

**IL-13 family**			
IL-3R	IL-3		Type 1
IL-5R	IL-5		
GM-CSFR	c-CSF, GM-CSF		
IL-3α	IL-3		
IL-5α	IL-5		
GM-CSFRα			

**IL-17 family**			
IL-17RA	IL-17A/IL-17F		Type 2
IL-17RB	IL-17B		
IL-17RC	IL-17C		
IL-17RD			
IL-17RE	IL-17E		
IL-25	IL-17B		

**TNF family**			
TNFR1	TNF		
TNFR2	TNF		

**IFN family**			
IFN-R, type 1	IFN-α, IFN-β, IFN-κ, IFN-ω		
IFN-R, type 2	IFN-γ		

*IL-1R type 2 is decoy receptor and binds IL-1α, IL-1β, and IL-receptor antagonist*.

*IL-1R family: possess immunoglobulin-like extracellular domains and an intracellular toll/IL-1R domain*.

*IL-2R: both α and β subunits bind ligand*.

*IL-6R: Ras/Raf/MAPK, PI 3 kinase/PKB*.

*IL-10: family of heterodimers*.

*IL-13R and IL-22Rα: there are also decoy receptors that bind IL-13 and IL-22*.

*IL-17 family: 6 ligands and 5 receptors*.

*Symbols α and β*.

*Heterodimers: IL-12A/IL-12B; IL-12B/IL-23*.

*Ra, receptor antagonist; TNF, tumor necrosis factor*.

Mature B cells express the CRs *BAFF-R* (highest affinity binding for BAFF), B cell maturation antigen (BCMA) or *BCMA* (highest affinity binding for APRIL, no BAFF binding), and transmembrane activator and calcium modulator and cyclophilin ligand interactor or *TACI* (BAFF and APRIL are both ligands) (28, 29).

Cytokine receptors may be *soluble* or cell membrane bound. Soluble CRs compete with cellular receptors for cytokine binding. sIL-27Rα, a naturally occurring soluble form of IL-27Rα, is an IL-27 antagonist produced by activated T cells, B cells, and myeloid cells (30). The soluble class II CR IL-22RA2 is a naturally occurring IL-22 antagonist (31). In the IL-1 cytokine family, there are naturally occurring inhibitors, such as IL-1 antagonists of IL-1R (19). There is also a soluble form of IL-6R resulting from cleavage by metalloprotein ADAM17 (32). There are also endogenous soluble receptors for TNF, IFN-γ, IL-2, and IL-4.

The TNF receptor family includes TNFR1 and TNFR2 receptors. TNFR1 is expressed on all nucleated cells, whereas TNFR2 is chiefly restricted to immune cells. Both membrane and soluble TNF-α activate TNFR1; TNFR2, mostly by mTNF-α (21). Genetic mutation of the TNF-R extracellular domain results fever and inflammation associated with TNF receptor-associated periodic syndrome.

The IFN receptor family includes receptors for type 1 (α, β, κ, and ω) and type 2 (γ) IFNs (26). The IFN-α receptor has 13 subunits: α1, α2, α4, α5, α6, α7, α8, α10, α13, α14, α16, α17, and α21 (26). IFN-R type 2, a decoy receptor, binds IL-1α, IL-β, and IL-1Ra (26).

### Chemokines

Chemokines or “*chemo*attractive cyto*kines*” are secreted proteins that govern leukocyte *trafficking* and *positioning* into targeted organs (33). They are well suited as biomarkers because they are most prevalent of human cytokines, have good performance characteristics, and can be correlated with clinical observations. Some are normal CSF constituents, while others are expressed in inflammation.

The newer structure-based nomenclature for CKs (34) has largely overtaken the older, descriptive nomenclature since introduced in 2000, but either are still used in the medical literature and can be compared (Table 2) (35). The naming comprises a prefix and number ID. CXCL10, for example, replaces “IFN-γ-inducible protein 10” (IFN-γ secretion is IL-12-family mediated) (36). C–X–C replaces the name α CKs; C–C, β CKs; C, γ CKs; and C–X–3C, δ CKs. CK ligands and their receptors comprise axes for chemotaxis of specific cell types.

**Table 2:** Chemokine structural and synonymous nomenclature.

Structural name	Acronym for alias	Common name/alias
**C–C motif**		
CCL1	I-309	
CCL2	MCP-1	Monocyte chemoattractant protein-1
CCL3	MIP-1α	Macrophage inflammatory protein-1α
CCL4	MIP-1β	Macrophage inflammatory protein-1β
CCL5	RANTES	Regulated on activation normally T cell expressed and secreted
CCL6	HCC-4	
CCL7	MCP-3	Monocyte chemoattractant protein-3
CCL8	MCP-2	Monocyte chemoattractant protein-2
CCL9	(Murine)	
CCL10	
CCL11	Eotaxin-1	Eosinophil chemotactic protein-1
CCL12	MCP-5	Monocyte chemoattractant protein-5
CCL13	MCP-4	Monocyte chemoattractant protein-4
CCL14	HCC-1	
CCL15	MIP-5	Macrophage inflammatory protein-5
CCL16	MTN-1	Monotactin-1
CCL17	TARC	Thymus and activation regulated chemokine
CCL18	MIP-4	Macrophage inflammatory protein-4
CCL19	MIP-3β	Macrophage inflammatory protein-3β
CCL20	MIP-3α	Macrophage inflammatory protein-3α
CCL21	SLC	Secondary lymphoid tissue derived cytokine
CCL22	MDC	Macrophage-derived chemokine
CCL23	MIP-3	Macrophage inflammatory protein-3
CCL24	Eotaxin-2	Eosinophil chemotactic protein-2
CCL25	TECK	Thymus expressed chemokine
CCL26	Eotaxin 3	Eosinophil chemotactic protein-3
CCL27	CTACK	Cutaneous T cell-attracting chemokine
CCL28	MEC	Mucosae-associated epithelial chemokine

**C–X–C motif**		
CXCL1	GRO-α	Growth-related oncogene
CXCL2	GRO-β	Growth-related oncogene
CXCL3	GRO-γ	Growth-related oncogene
CXCL4	CD184	(Platelet-derived chemokine)
CXCL5	(Murine)	
CXCL6	GCP2	Granulocyte chemotactic protein 2
CXCL7	TCK-1	Thymus chemokine-1
CXCL8	IL-8	Interleukin-8
CXCL9	Mig	Macrophage inflammatory protein-1γ
CXCL10	IP-10	Interferon-γ-inducible protein-10
CXCL11	I-TAC	Interferon-inducible T cell alpha chemokine
CXCL12	SDF-1α	Stromal cell-derived factor-1 alpha
CXCL13	BCA-1	B cell attractant-1
CXCL14	MIP-2γ	Macrophage inflammatory protein-2γ
CXCL15	Lungkine	
CXCL16	ROCK1	Rho associated coiled-coil containing protein kinase-1
CXCL17	GPR35	G protein-coupled receptor ligand 35

**C- motif**		
XCL	Lymphotactin	

**C–X–3C motif**		
CX3CL1	Fractalkine	

*Reference (Zlotnik00)*.

C–C ligands (1–28), the largest group of CKs, correspond to C–C receptors (CCRs), just as C–X–C CKs (1–17), the second largest CK subgroup, do to C–X–C receptors (CXCRs), and there is functional relatedness between subgroup ligands (7). XCL (two types) and CX3C (one type) are smaller ligand subgroups. Different CKs are involved in the trafficking of different kinds of immune cells, such as neutrophils (CXCL1–8), monocytes (CCL1–5, 7, 8, 13–16, 18, 23), T cells (CXCL9–11), B cells (CXCL13, CXCL10), and monocytes/T cells [C–X–3C chemokine ligand motif (CX3CL1) or fractalkine] (22). CKs also have been stratified as to whether they are lymphoid CKs (CXCL12, CXCL13, CCL19, and CC21) or inflammatory CKs (CCL2, CXCL9, CXCL10, and CXCL11) (9).

“Homeostatic” CKs (CCL14, CCL19, CCL20, CCL21, CCL25, CCL27, CXCL12, and CXCL13) are also involved in the maintenance of *homeostasis* (12). During brain development, migration, proliferation, and differentiation of neural progenitor cell migration is guided by CXCL12 and CXCL14. *Immunosurveillance* involves immune cell trafficking in response to CCL2, CCL19, CCL20, CCL21, and CXCL12 (12). CX3CL1, which is expressed on neurons, is pivotal to maintenance of *immune quiescence*; the receptor is present in the CNS only on microglia (7). “Mucosal CKs” encompass CCL25, CCL28, CXCL14, and CXCL17 (37). “Angiogenic CKs” comprise many of the CXC CK family, such as CXCL1 through CXCL17 (38).

#### Chemokine Receptors (CKRs)

Classical or typical CKRs are seven membrane G protein-coupled-receptors (GPCRs), which have been the most successful target class for drug discovery (39). Their expression may be constitutive or inducible. They participate in ligand–receptor pairs or axes, with CKs functioning as receptor agonists (full, partial, and inverse) or antagonists as standardly defined (40). There are “major” and “minor” receptor “pockets” to which CKs bind, resulting in different functionalities. The domain of CKs comprises roughly 50 peptides and 20 receptors in humans (11), indicating that several ligand–receptor relationships are not exclusive (“promiscuous”). CKRs for inflammatory CKs are especially promiscuous, some lacking an endogenous CK (41). Those that are exclusive involve “cognate” receptors. Most CKs signal through GPCRs, targeted by about 30% of current pharmaceuticals, which have been called the most “druggable” receptor class (42). The repertoire of receptor expression depends on the type of leukocyte and its maturational stage (42). The receptors may bind CKs which exhibit opposite roles: CXCR7 binds CXCL12 and CXCL11 (39). While CKR groupings may be cell specific, CXCR4 is expressed by all immune cells. Collectively, these factors confer the differential responsiveness of target cells.

Recently, “atypical chemokine receptors” (ACKRs)—without GPCR signaling or chemotactic effects—that scavenge CKs at inflammatory sites have been described (43). ACKRs, formerly termed “decoy” or “scavenging” receptors based on their capacity for sequestering cognate CKR ligands, are important for CK regulation (44). They were also referred to as “silent” receptors, based on lack of classic CKR signaling and functional activities like directly inducing immune migration of immune cells (45). ACKRs are now conceptualized as “endogenous β-arrestin-biased signaling receptors” and bind multiple CKs (45). Most ACKRs are expressed on vascular endothelial cells; some have additional strategic locations: Purkinje cells (ACKR1), B1 B cells (ACKR2), and neurons (ACKR3) (44).

**Figure 1:**
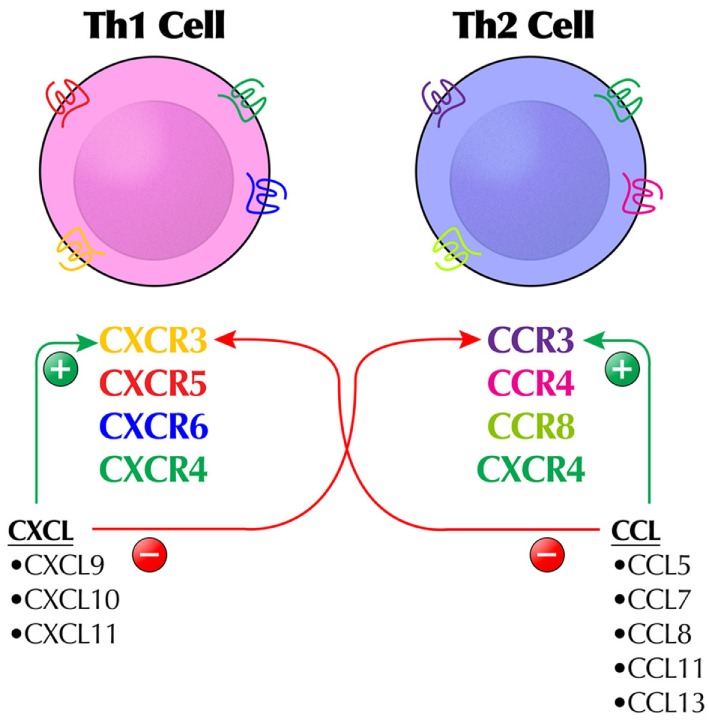
The complex regulation of the chemokine (CK) system by a combination of agonist and antagonist activity illustrates why some CKs or chemokine receptors are not easy targets for immunomodulation and how different immunologic outcomes may occur. For example, CXCL9–11 are agonists at CXCR3 (expressing Th1 cells), but antagonists at CCR3 expressed by Th2 cells. Likewise, CCL5, CCL7, CCL8, CCL11, and CCL13 are agonists at CCR3-expressing Th2 cells. The typical stylized receptor depiction is meant to show the extracellular and intracellular domains of the receptor, which is a seven-transmembrane receptor.

Most typical human CKs (C–C or C–X–C) are CKR agonists according to the IUPHAR classification (Table 3) (41, 42). A promiscuous CK may function exclusively as an agonist at more than one CKR, such as CCL2 (CCR2, CCR3, and CCR5); only as an antagonist at more than one receptor, such as CCL18 (CCR1 and CCR3); or as an agonist at one receptor and an antagonist at another, such as CCL4 (agonist at CCR5, antagonist at CCR1) (Figure 1). Certain CK ligands are agonists at both typical CKR and ACKR, such as CCL19 (CCR7, CXCR3, and ACKR4) or CXCL12 (CXCR3, CXCR4, and ACKR3). Receptor conformations on leukocytes are particularly important, potentially resulting in blocking signaling by one conformation but not the other. CCL17 and CCL22 both bind to CCR4, but to different conformations, although both can evoke CCR4 internalization (46). Many ACKRs are not yet specified as to agonist/antagonist status.

**Table 3:** Human CKR agonists and antagonists.^a^

CKR	C–C–L agonists	C–C–L antagonists
**CCR-**		
1	3, 5, 7, 8, 13, 14, 15, 16, 23	4, 18
2	2, 7, 8, 11, 13, 16	24, 26
3	2, 5, 7, 8, 11, 13, 15, 28	18 (also CXCL9, 10, 11)
4	17, 22	
5	2, 3, 4, 5, 8, 11, 13, 14, 16	7
6	20	
7	19, 21	
8	1	
9	25	
10	27, 28	
**CXCR-**		
3	5, 13, 19, 20	7, 13
**ACKR-**		
4	19, 21, 25	

**CXCR-**	**C–X–C agonists**	**C–X–C antagonists**

1	1, 2	
2	1, 2, 3, 5, 6, 7, 8	
3	9, 10, 11, 12	
4	12	
5	13	
6	16	
**CX3CR1-**		
6	CX3CL1	
**XCR-**		
1	XCL1	
**ACKR-**		
3	CXCL11, 12	

*^a^Per the International Union of Pharmacology designations (41). The numbers in the left column are the receptor numbers (CCR1, CCR2, etc.), and the numbers under the agonist and antagonist columns are the ligand numbers (CCL3, CCL5, etc.)*.

*CKR, chemokine receptor; CCR, C–C–R motif chemokine receptor; ACKR, atypical chemokine receptor; CXCR, C–R motif chemokine receptor; CX3CR, C–X3–C–R motif chemokine receptor; XCR, X–C–R motif chemokine receptor*.

The concept that CKRs may serve as biomarkers in neuroinflammatory disorders has been proffered (47). CK/CR-expressing immune cells can be measured *ex vivo* through flow cytometry (5). T cells in the CSF express a delimited array of inflammatory CKRs: enrichment of CCR5, CCR6, CXCR3 (little CCR1, CCR2, and CCR3) independent of the presence of neuroinflammation (48). The cellular and spatial distribution of CKRs in the brain has been reviewed (49). CXCR2 (and ligand CXCL1) plays a developmental role in *gliogenesis* (7). Ties between the nervous and immune systems are well illustrated by the fact that gamma-aminobutyric acid (GABA)-A and GABA-B agonists/antagonists are allosteric modulators of CXCR4.

### Cellular Sources of Chemo/Cytokines: Leukocyte Trafficking and Positioning

Because excessive recruitment of leukocytes to inflammatory sites is a hallmark feature of inflammatory disorders, specific inhibition of this recruitment would be an ideal anti-inflammatory strategy.

The initial approach to the study of leukocyte trafficking to the CNS is descriptive analysis of CKs in CSF (and peripheral blood), and CKRs expressed by cells circulating in these compartments (Table 4) (6, 7, 10–13, 25, 33, 34, 39, 47, 49–52). Hematogenous cells may enter the CNS through different barriers, such as blood–CSF and blood–brain (7). In neuroinflammatory disorders, pro-inflammatory cytokines are mainly secreted by invading leukocytes, whereas in neurodegenerative diseases they are produced principally by CNS-resident cells (innate immunity) (6). The process is *dynamic*: immune cells follow the gradient of CKs (also called “chemotactic factors”). CK concentrations in circulating blood are miniscule (10^−12^ M), but can increase 1,000-fold. Immune cells upregulate or down regulate their CKRs accordingly.

**Table 4:** Chemokine receptor (CKR) expression and cytokine production by type of immune cell.

Adaptive immune cells (hematogenous, CNS invading)								
T cells^a^					B cells		Other cells	
Th1	Th2	Th17	Tfh	Treg	B	Breg	DC^b^	NK^b^^,^^c^
**Receptors expressed^d^**								
CXCR3	CCR3	CCR6	CXCR5	CCR4	CXCR5	CXCR5	CCR7	CXCR3
CCR5	CCR4	CCR4		CCR6		CCR6	CCR5	CXCR1
CXCR6	CCR8	CCR2					CCR6	CCR7
CCR2		CXCR3					CXCR3	CCR2

**Cytokines secreted^e^**								
IFN-γ	IL-4, 5, 6	IL-17A, F	IL-21	IL-10	IL-5, 10	IL-10	IL-23	IFN-γ
IL-2	IL-13, 25	IL-21, 22, 23	CXCL12	IL-35	IL-12, 14	IL-35	IFN-α	TNF-α
IL-12p70	IL-31, 33	IL-1β, 6, 8	CXCL13	TGF-β	TNF-β	TGF-β	IL-12	
TNF-β	CCL21	G-CSF						
CXCL9, 10, 11		GM-CSF						

**Innate immune cells (CNS resident)**								

**Neurons**	**Astrocytes**	**Microglia**	**Vascular endothelial**		**Macrophage (M1)**		**Macrophage (M2)**	

**Receptors expressed**								
CXCR7^a^	CX3CR1	CX3CR1	CXCR7		CX3CR1		CX3CR1	
	CCR2, 3, 5	CCR2						
	CXCR2–4	CCR5						
		CXCR3						

**Cytokines secreted**								
CX3CL1	BAFF	IL-1	CCL19		IL-1α, β		IL-4, 10, 13	
IL-34	CXCL10	IL-6	CCL20		TNF-α		TFG-β	
CCL2	CCL2	TNF-α	CCL21		IL-6, 12, 23			
CXCL14	IL-1, 6, 10, 15, 27	TGF-β			CCL2			
	TGF-β				CXCL9, 10			

*Th, T helper cell; Thf, T follicular helper cell; Treg, regulatory T cell; Breg, regulatory B cell; DC, dendritic cell; NK, natural killer; CCR, C–C motif chemokine receptor; CXCR, C–X–C motif chemokine receptor; IL, interleukin; IFN, interferon; TNF, tumor necrosis factor; TGF, transforming growth factor; C-CSF, granulocyte colony-stimulating factor; GM-CSF, granulocyte–monocyte colony-stimulating factor; CNS, central nervous system*.

*Some CKRs are expressed only under certain conditions*.

*NKT cells (not shown) express CCR1, CCR2, CCR4, CCR5, CXCL13, and CXCR6*.

*^a^γδ T cells (not shown) express CXCR3 and CCR5 CKRs and secrete IL-17*.

*^b^DC subtype cytokine secretion: plasmacytoid DC, IFN-α; myeloid DC, IL-12*.

*^c^NK cell subtypes express different CKRs: CCR7 on CD56bright, not dim NK cells*.

*^d^CXCR4 is expressed constitutively by all of the cells, does not differentiate them, and is therefore not shown, although important*.

*^e^Groupings are based on typical, most abundant cytokine products and effector functions and are not exhaustive. Cytokines may play opposite roles under some circumstances and may be classified under alternative Th roles. Multiple Th cells secrete cytokines TNF-α, GM-CSF, IL-9, IL-15, IL-27, IL-28A, IL-31, IL-12 (p40), IL-12 (p70), IL-1Ra, and IL-1β and chemokines CCL2, CCL3, CCL4, CCL21, and CXCL1*.

“Conversing” or *signaling* between T cells infiltrating the CNS and resident microglia, astrocytes, and other members of the innate immune system is important to the discussion of cellular sources of chemo/cytokines (25). CNS immunity has both *afferent* and *efferent* limbs. DCs, part of innate immunity, are components of the afferent limb, while central-memory T cells, entering the CSF from the circulation execute the efferent limb (53).

The response of the immune system to injury is sequential. Alarmin release and glial activation is followed (in minutes to hours) by inflammasome activation, cytokine production, and neutrophil recruitment, infiltration of monocytes (hours to days post injury), then recruitment of adaptive immune cells (days to weeks post injury) (54).

#### Infiltrating Immune Cells

*T cell subsets* produce “signature cytokines” in inflammatory disorders (55). CD4+ lymphocytes comprise *effector* and *regulatory* cells. While classic *T helper (Th) cell* Th1 and Th2 phenotypes have been long studied, effector CD4+ T cells can be further classified based on immunological function and other characteristics. Th1 and Th17 cells play a role in neuroinflammatory disorders; Th2 cells, in allergy (55). However, the plasticity of Th17 cells allows them to shift toward a Th1 or Th2 phenotype, displaying more aggressive or pathogenic actions than unshifted cells (56). Th1 cells (IFN-γ and TNF) are largely responsible for cellular immunity; Th2 cells (IL-5, IL-5, and IL-10), for humoral immunity. Th0 cells may also differentiate into Th17 cells (IL-6 and IL-17), Th22 cells (IL-22), Th9 cells, and follicular helper cells (Tfh) (21, 56). Atypical T cells include gamma/delta T cells and natural killer-like (NKT) cells. In the CNS, γδ T cells comprise two subsets with opposing roles. The Vγ4+ subset secretes IL-17 and other pro-inflammatory cytokines, whereas the Vγ1 subset produces CCR5 ligands (57).

*B cell* trafficking, differentiation, and survival have been better understood as a result of clinical application of anti-B cell monoclonal antibodies to human neuroinflammatory disorders, establishing a role for B cells in many, such as multiple sclerosis (MS) (40, 58). CXCL13, the preferential chemoattractant for B1 cells, plays a particular role in B cell trafficking and homing, and its CSF:serum ratio is a measure of chemotactic gradient (58). CXCR5, the cognate receptor for CXCL13, is expressed on almost all B cells, more so on B1 (T cell-independent) than B2 (T cell-dependent) cells. CXCR5 is also expressed by follicular helper CD4+ T cells (Tfh), which provide T cell help to B cells (59).

*Dendritic cells*, the most powerful professional antigen-presenting cells, play a pivotal role in initiating and perpetuating CNS-directed autoimmune responses involving B and T cells (60). The CNS lacks resident DCs, thereby conferring “immune-privilege” (7), so they operate at the interface of the adaptive and innate immune systems. CCR7 controls the cytoarchitecture, rate of endocytosis, survival, migratory speed, and maturation of DCs, modulating the immune response by regulating different functions of DCs. An important ligand for CCL7 is CCL21. DCs are a major source of CCL22 and CCL17. Also, activated T cells strongly upregulate CCR7 and migrate to CCL19.

*Natural killer cells* also use CSF as an intermediary compartment for trafficking into the CNS and for differentiation (61). CSF NKs exhibit reduced CX3CR1 expression compared with blood NKs. In blood, CD56dim CD16+ cells, which are cytotoxic and secrete more cytokines in response to IL-2, predominate. CKs play an important role in the phenotype and functionality of resting (CD56dim) vs activated (CD56bright) NK cells (62).

*Monocytes* invading the CNS transform into inflammatory macrophages or DCs (6). The chemoattraction of monocytes to inflammatory sites owes to their expression of CCR2 and corresponding ligand CCL2 (63). Monocytes, macrophages, and DCs together comprise the mononuclear phagocyte system (6).

Other cells include mast cells, first responders able to initiate and amplify CNS immune responses and secrete cytokines, and neutrophils (especially in bacterial meningitis), but will not further discussed.

Various infiltrating immune cells participate in *lymphoneogenesis* in the CNS. The CXCL13/CXCR5 ligand/receptor pair (like CCL21/CCR7 and CXCL12/CXCR4) has the capacity to induce the ectopic lymphoid-like tissue, common to chronic autoimmune diseases, thereby maintaining B and T cells in lymphocytic infiltrates. Inflamed brain can become a niche for B cell survival and proliferation through BAFF and B cell-activating factor receptor expressed on B cells. Chemo/cytokines linked to lymphoid neogenesis in the CNS include CXCL13, CXCL12, CXCL10, IL-6, and IL-10 (64).

#### Innate Immune Cells

The concept of bidirectional *neuron–glia communication via* CKs and other cytokines, one the one hand, and neurotransmitters and neuropeptides, on the other, is central to understanding physiological functionalities and pathological distortions. Certain inflammatory CKs cause synaptic hyperexcitability by altering glutamatergic and GABAergic neurotransmission and affect neuroplasticity (65).

Central nervous system-resident cells (neurons, astrocytes, microglia, oligodendrocytes, and endothelial cells) are sources of CKs (6), as demonstrated in postmortem studies of MS. Non-microglial myeloid cells include macrophages (choroid plexus, perivascular, and meningeal) and DCs (6). Two important categories of cell signaling (cross talk) are between neurons and microglia and between microglia/astrocytes and CNS-infiltrating cells (25).

*Astrocytes*, the most abundant non-neuronal cells in the brain, interact with neurons in brain development and function, providing anatomic support, such as end feet surrounding capillaries in the BBB, and physiologic support, including modulation of synaptic activity and blood flow, and regulating water, potassium, and glutamate in the CNS (25). As to the immunologic functions, CCL2/CCR2 is important for astroglial–neuronal signaling. CXCL12 and CX3CL1 (exclusively on microglia and astrocytes) are constitutively expressed. CCR3, CCR5, and CCR7, as well as CXCR2, CXCR3, and CXCR4 are constitutively expressed by astrocytes. In response to injury, astrocytes can secrete CXCL10 and BAFF, but also anti-inflammatory cytokines like IL-10. CX3CL1 and IL-34 are neuronally produced “help me” signals (42). In MS plaques, BAFF, CCL2, and CXCL10 are upregulated near hypertrophic astrocytes (50, 66). In surgical specimens of children with Rasmussen encephalitis (RE), neurons and astrocytes in damaged areas expressed CXCL10, and cytotoxic infiltrating T cells in the same area expressed CXCR3 (67). Astrocytes are the major source of IL-6 in the CNS and may help regulate synaptic function (68).

*Microglia* are the only lifelong resident immune cells of the CNS (25). Microglia precursors migrate early from the yolk sac to colonize developing brain (69). Other glial cells are neuroectoderm derived (51). High, not low, levels of CXCL12 activate CXCR4 on microglia, initiating TNF-α release, which causes astrocytes to release glutamate (excitotoxicity). CX3CL1/CX3CR1 is key to neuronal–microglial signaling after injury: neurons secrete CX3CL1, but CX3CR1 is expressed only on astrocytes and microglia. Microglia are activated by chronic neurodegeneration and systemic inflammation (25). Microglial–astrocyte interactions are vital to innate immunity in the CNS. IL-6R is present in microglia, not astrocytes (70).

Activated astrocytes and microglial cells release neuroactive “gliotransmitters” *via* exocytosis, akin to neurotransmitter release from nerve endings (71), and alter neuronal chloride (72). They modulate blood–brain barrier permeability and BBB-mediated neuroinflammation and affect cell-to-cell communication (73). Gliotransmitters include GABA and taurine (inhibitory gliotransmitters) (72), as well as glutamate, d-serine, ATP, and l-lactate (71).

*Macrophages* derived from invading monocytes are the predominant cell type presenting antigen to infiltrating T cells during neuroinflammation (6). Myeloid cells are the principal source of cytokines influencing T cell effector functions (74). The M1/M2 macrophage classification may not apply to CNS resident macrophages (6).

#### Endothelial Barriers

The homeostatic CKs expressed on the endothelial barriers include CXCL12, CCL19, CCL20, and CCL21 (12). DCs are proximal to the choroid plexus and meningeal blood vessels. The endothelium of the blood–brain barrier expresses CXCR4 and CXCR7, a scavenger receptor for CXCL12. CKs are presented on endothelial cells *via* binding to glycosaminoglycans (GAGs), which is required for leukocyte migration. CNS barriers have been reviewed extensively (10, 12, 25).

### Signaling and Transduction Mechanisms of CK/Cytokine Actions

Intracellular chemo/cytokine signaling cascades are the focus of intensive research. As GPCRs, CKR couple to transducers, such as heterotrimeric G proteins and β-arrestins, which then engages intracellular signaling cascades (75). These result initially in desensitization and receptor internalization. The subsequent intracellular signaling cascade may involve activation of nuclear factor kappa-light-chain-enhancer of activated B cells (NF-κB), extracellular signal-regulating kinase (ERK), mitogen-activated protein kinase (MAPK), extracellular signal-regulated kinase (ERK), phosphatidylinositol-3 kinases, Janus kinase (JAK)/signal transducer and activator of trascription (STAT), or other proteins (76). Transcription of genes encoding IL-1 family cytokine precursor proteins (pro-IL-1β and pro-IL-18) occurs in response to tissue injury or infection, resulting in inflammatory cytokine production *via* the inflammasome (25). IL-1β is activated when enzymatically cleaved by activated capsase-1. Signaling and transduction involves JAK1, 3/STAT5 for IL-2; JAK2/STAT5 for IL-3, and glycoprotein 130 (gp130)/JAK/STAT for IL-6 (26). Not all chemo/cytokine signal transduction results in inflammation. A case in point is IL-37, a natural suppressor of inflammatory responses, which transduces anti-inflammatory signaling *via* suppression of NF-κB and MAPK (77). New therapeutic strategies may capitalize on the negative regulation of the NF-κB pathway (78). *Biased signaling* (or biased agonism) refers to the capacity of CKR to activate a particular signaling pathway from among others in a biased manner. Further commentary is deferred to other reviews (6, 7, 12–14).

## Comparison of CSF CK/Cytokine Immunomarker Profiles in Human Neuroinflammatory Disorders

Strategizing how to target CKs and other cytokines relies on knowing which, if any, are part of the intrathecal immune response in a particular disease. The unique modality of profiling CSF CKs and other cytokines reveals the presence of immune *dysregulation*, or disturbed homeostasis (33). Proper evaluation of the CSF depends on reliable measurement of the biomarkers, normal ranges per patient age, and knowledge of the test limitations. First, it should be recognized that not all cytokines are equally detectable (Figures S1 and S2 in Supplementary Material and its legends). In CSF, CKs are found in greater concentrations than other cytokines. CXCL10 had the highest concentration, and CCL2, CXCL10, and IL-6 were detectable in all samples (35). Other CSF cytokines also are measurable, but their concentrations are extremely different. In children with non-inflammatory neurological disorders (35), the percent detectability was highest for IL-16, IL-1Ra, and IL-6, declining sharply for the others. Calculation of the CSF/serum or CSF/plasma chemo/cytokine gradient is not always reported, yet it is crucial for making inferences about intrathecal production and CNS compartmentalization of the immune response (79–81).

### Disease Effects on Individual CSF Immunomarkers and Group Patterns in Non-Infectious Disorders

With the methodological advent of simultaneous measurement capability of multi-analyte CKs/cytokines in a small volume of CSF obtained by lumbar puncture (LP), there are now more studies of soluble CSF CKs/cytokines in various neuroinflammatory disorders (Table 5) (3, 9, 16, 17, 50, 52, 59, 79–121). Many are disorders studied in adults, but there has been an upgrowth of pediatric investigations, and with them the increasing availability of pediatric reference ranges (35, 52). Cameos of the pediatric-onset disorders can be found in the Table S1 in Supplementary Material.

**Table 5:** CSF chemo/cytokine biomarker signatures in key neuroinflammatory disorders.

Disorder (*n*)	Cytokines						CKs				
	IL-				TNF family		CXCL-			Other ↑	Reference
	6	8^a^	10	17	TNF	BAFF	10	12	13		
**Demyelinating**											
RR-MS (28)	↑	↑	↑	↑	↑	↑/↔		↑/↔	↑	IL-12p40, IL-13	(3, 50, 59, 82, 83)
										CCL19	
“MS” (20)	↔	↔	↔	↔				↔		CCL5, IL-2Ra, CXCL1, CXCL1	(84)
SP-MS (12)						↔	↔	↔	↔		(9)
*pMS (17)	↑								↑		(85)
*pMOG(+)^e^	↑		↑	↑	↑			↑		CCL19, APRIL	(86)

NMO (21/9)	↑	↑	↑	↑	↑	↑	↑		↑	G-CSF, IL-13, IL-1Ra, IL-21	(82, 87–89)
										IL-1β, CCL11, APRIL	
								(↓)			(90)

ADEM (14/17)^b^	↑	↑	↑	↔	↑/↔		↑			G-CSF, IL-18, IL-2, IL-5, IFN-γ	(16, 91)
										CCL1, 3, 5, 17, 22	
										CXCL1, 7	
*pADEM (11)	↑		↑	↑	↑		↑	↔	↑	IFN-γ, IL-4	(52)
										IL-21, CXCL9, CCL19	

AIDP (22)	↑			↑			↑			IL-22, CCL2	(92)
pM–F	↑	↑									(93)
CIDP (/24)^d^	↑	↑		↑			↑			CCL2, CCL19	(17)
										IL-12	

**Paraneoplastic/encephalitic**											
*pOMS (239)	↑	↔	↔	↔	↔	↑	↑	(↓)	↑		(79–81)
(17)						↑					(94)
pAnti-Hu (1)						↑	↑	(↓)	↑		(95)
AE (27)	↑			↑	↑	↔	↑	↑	↑	IFN-γ, IL-15	(96, 97)
*pAE (16)	↑		↑	↔	↑		↑	(↓)	↑	IFN-γ, CXCL9	(52)
*pSC (14)			↑							IL-4	(98)
PCD		↔					↑				(99)

**Epileptic**											
*pFIRES (1)	↑	↑	nd			Nd		↔			(100)
*pRE (27)^f^				↑							(101)
*pIS (12)	↓/↔										(92)
(24)										(↓ IL-1Ra)	(103)
AGS	↔	↔					↑			CCL2, IFN-α	(104)

**Vascular**											
NPSLE (52/42/28)	↑	↑			↓	↑	↑	↑	↑	G-CSF, GM-CSF, CCL2, CCL4, CCL5, APRIL	(105–107)
*pNPSLE (1)	↑	↑				↑	↑	↑	↑	CCL19	(108)

N-Beçhet (68)	↑										(109)
pN-Beçhet (1)	↑										(110)

Stroke (30)	↑	↑	↑				↑			GM-CSF, CXCL1, CCL2, CXCL5	(111, 112)

**Other neuroinflammatory**											
*pNOMID (17)	↑						↑			IL-18	(113)

*pTBI	↑	↑	↑							IL-1β, CCL3, sIL-2R	(114)
(105)^g^	↑									IL-12	(115)

pPHH (11)			↑							IL-1α, IL-1β, IL-12, CCL3, CCL19	(116)
SS-myelitis	(9)									CCL3, CCL4	(117)
pSS-ME (1)	↑				↔						(118)
TM	↑	↑	↑	↔							(119)

**Neurodegenerative/IEM**											
SCA (12)^c^	↑	(↓)	(↓)	↔	(↓)		↑			IL-7, IL-9, IL-12, IL-13, GM-CSF	(120)
MSA-C (20)	↑	(↓)	(↓)	↔	(↓)		↔			IL-7, IL-12, IL-13	(120)
*p/aMLD (8)		↑								CCL2, CCL4, IL-1Ra	(121)
pALD		↑								IL-1ra, CCL2, CCL4	(122)

*p, pediatric; *p/a, mixed pediatric and adult; ↑, increased concentration; ↓, decreased concentration; ↔, normal concentration; nd, not detected.

*RR-MS, relapsing–remitting multiple sclerosis; SP-MS, secondary-progressive MS; MOG, myelin oligodendrocyte glycoprotein; NMO, neuromyelitis optica; ADEM, acute disseminated encephalomyelitis; AIDP, acute inflammatory demyelinating polyradiculoneuropathy or Guillain–Barré syndrome; CIDP, chronic inflammatory demyelinating polyneuropathy; OMS, opsoclonus–myoclonus syndrome; M–F, Miller–Fisher syndrome; Anti-Hu, Hu or ANNA-1 antibody paraneoplastic syndrome; AE, anti-NMDAR encephalitis; SC, Sydenham chorea; PCD, paraneoplastic cerebellar degeneration; FIRES, febrile infection-related epilepsy syndrome; RE, Rasmussen encephalitis; IS, infantile spasms; AGS, Aicardi–Goutières syndrome; NPSLE, neuropsychiatric systemic lupus erythematosus; N-Beçhet, neuro-Beçhet disease; NOMID, neonatal-onset multisystem inflammatory disease; TBI, traumatic brain injury; pPPH, post-hemorrhagic hydrocephalus; SS-ME, Sjögren syndrome meningoencephalitis; TM, transverse myelitis; IEM, inborn error of metabolism; SCA, spinocerebellar atrophy; MSA-C, multiple system atrophy cerebellar type; MLD, metachromatic leukodystrophy; ALD, adrenoleukodystrophy (males); BAFF, B cell-activating factor; APRIL, a proliferation-induced ligand; CK, chemokine; CSF, cerebrospinal fluid; GM-CSF, granulocyte–monocyte colony-stimulating factor; MS, multiple sclerosis; TNF, tumor necrosis factor*.

*^a^IL-8, CXCL8; CCL, C–C motif CK ligand; sIL-2R, soluble IL-2 receptor*.

*^b^Adult ADEM: ↑ IL-6 and CKs (not Th1/Th2 cytokines IL-2, IL-4, IL-10, TNFα, and IFNγ—they were low) (58)*.

*^c^SCA and MSA-C: decreased TNF-α, IL-1β, IL-2, IL-15, IL-4, and IL-5 (80)*.

*^d^Adult CIDP: decreased IL-4, IL-5, and IL-7 (66)*.

*^e^Did not include MS*.

*^f^RE: normal CSF IL-4, IL-12, and IFN-γ; increased granzyme B*.

*^g^Ventricular CSF from intraventricular catheter on continuous drainage*.

Aggregate observations culled from the table provide evidence of dysregulated expression of CNS CKs and other cytokines and indicate several trends. First, CSF IL-6 was increased in all the presented disorders, and CXCL10 and CXCL13 were found to be elevated when they were measured. Second, both *pro-inflammatory* and *anti-inflammatory* cytokines (IL-10, IL-4, IL-5, and IL-13) were present in most disorders studied, but the IL-10 concentration was reduced in the degenerative disorders spinocerebellar atrophy (SCA) and MSA-C. Third, most intrathecal inflammatory mediators were found to be increased; however, concentrations of a few were decreased or unchanged. The concentration of CXCL12 was decreased in pOMS, *N*-methyl-d-aspartate receptor (NMDAR) encephalitis, pAnti-Hu/ANNA-1 paraneoplastic syndrome, and neuromyelitis optica (NMO), but increased in pMOG, adult AE, and neuropsychiatric systemic lupus erythematosus (NPSLE). The decrease may reflect CXCL12 relocation to the blood–brain barrier, decreased production, or increased consumption (81). CSF TNF-α was decreased in NPSLE, normal in pOMS, and increased in several other disorders. Fourth, while inflammatory markers raged in CSF, serum concentrations did not reflect it in NPSLE (106), pOMS (79–81), or stroke (112). Fifth, other than for MS, there have not been multiple studies of other disorders to compare with each other. One line of evidence that these immune mediators are operatives in MS is the marked decline in CSF concentrations of CKs (CXCL9, CXCL10, CXCL11, and CCL22) and other cytokines (IL-1β, IL-6, and IL-8) following natalizumab treatment (123).

Cerebrospinal fluid abnormalities listed in the “Other” column warrant mention, although data were fragmentary due to not being systematically measured, thereby limiting between-disease comparisons. Inflammatory cytokines prevailed: INF-γ [acute disseminated encephalomyelitis (ADEM), pADEM, AE, pAE], IL-12 [relapsing–remitting multiple sclerosis (RR-MS), chronic inflammatory demyelinating polyneuropathy (CIDP), pTBI, SCA, MSA-C, G-CSF (NMO, ADEM, and NPSLE), and GM-CSF (stroke, NPSLE, and SCA). The concentrations of BAFF and APRIL were mostly congruous, both usually being elevated (NMO and NPSLE), with a few notable exceptions (pOMS). The only two elevated CKs were CCL19 (RR-MS, pMOG, pADEM, and CIDP) and CCL2 (acute demyelinating polyradiculoneuropathy, NPSLE, CIDP, metachromatic leukodystrophy, and adrenoleukodystrophy).

There are also quantitative differences in the concentration of a particular CK/cytokine between neuroinflammatory disorders, best appreciated when more than one disorder is compared in the same study. Usefulness of CSF CK quantitation as a diagnostic aid in children with neuroinflammatory disorders has been advocated (124); however, no attempt was made here to denote quantitative differences between studies, because differences in biomarker sensitivities related to kits and laboratories would confound the comparison. The heterogeneity, variability and inconsistencies of chemo/cytokine concentrations in different studies in the same neuroinflammatory disorders may make it difficult to corroborate results and see unifying patterns due to variable severity of diseases, timing of CSF samples, prior treatment, and different assay methods. Some markers are investigated only sporadically.

Concentrations of immune markers may or may not correlate with those of diagnostic autoantibodies or CSF oligoclonal bands. CSF IL-6 correlated with anti-myelin oligodendrocyte glycoprotein (MOG) antibody titers (86), but only weakly with anti-aquaporin-4 antibodies (88). CSF IL-6 concentration, which is increased in monophasic acquired demyelinating syndromes more than in MS, correlated with plasma MOG antibodies (86). In anti-NMDAR encephalitis, elevated CSF CXCL13 correlated with intrathecal NMDAR-antibody synthesis (96).

Cerebrospinal fluid CKs/cytokines that correlate with disease severity or outcome are perhaps the most promising from the standpoint of clinical immunotherapeutics. In pOMS, CSF CXCL13 concentration correlated with motor severity and disease duration (81). In severe pediatric traumatic brain injury (TBI), CSF cytokines/CK concentrations did not correlate with the initial score on the Glasgow Coma Scale or outcome (114).

Various combinatorial panels of immunobiomarkers have been proposed as best able to reveal active intrathecal inflammation. One is the combination of CSF IL-8, IL-12p40, and CXCL13 (3). Another is TNF-α, IFN-γ, IL-6, IL-10, CXCL10, and CXCL13 (52). The combination of IL-2, IL-17, IFN-γ, IL-5, FGF-basic, and IL-15 was able to distinguish NPSLE from MS and NMO (107). Given the potential combinatorial diversity, other panels are being explored.

Other small studies also have been reported, but with pitfalls in interpretation (1). In a study of Aicardi–Goutières syndrome (AGS), multiple CSF cytokine and CK abnormalities were shown (125), but would not have been significant if correction for multiple comparisons had been made. In complex disorders, there may be a need for multiple active controls, such as seizures, movement disorders, fever, which may not be provided. Or the active controls may be provided, but without passive controls (102). Studies of one disease compared with another without passive controls (126) can only be interpreted in light of reference ranges that share similar methodology. Immunobiomarker studies in uncommon disorders are challenging but critical.

It is customary to differentiate “primary” vs “secondary” neuroinflammatory disorders, but the defining line is blurring. Comparison of the CSF profile of TBI or ischemic stroke, in which neuroinflammation is secondary, with RR-MS, in which neuroinflammation is presumed to be primary, is not revealing of the difference, based at least on the markers studied. More autoantibody-mediated neuroinflammatory disorders are being identified in the aftermath of CNS infections. Also, neuroinflammation may lead to neurodegeneration (66). Leukodystrophies are the most common cause of pediatric neurodegeneration (127), and in one of them (Krabbe disease), recent studies show that immune activation precedes clinical symptoms and pathology (127).

Cytokines and CKs also may aid in differential diagnosis of diagnostically undefined cases by revealing active intrathecal inflammation. One need involves being able to predict whether the clinically isolated syndrome (CIS) will progress to MS. CSF CXCL13 (128) and IL-12p40 (129) are characteristic of MS, not CIS. Subclinical central inflammation marked by CXCL8, but not IL-17, is also a risk for CIS conversion to MS (130).

### Effects on CKR Expression

Cerebrospinal fluid CK/cytokine perturbations are best interpreted with knowledge of corresponding receptor expression on immune cells. This requires flow cytometry on freshly collected immune cells, a different technology than the immunomarker kits, so is less often performed. CKR profiling (Table 6) (48, 53, 80, 81, 131–133) has revealed several active CK ligand–receptor axes involving T cells and B cells in MS and pOMS. For example, the significance of an elevated concentration of CXCL13 is interpretable with the finding that memory B cells expressing the cognate receptor for CXCL13 (CXCR5) are expanded in frequency in the CSF.

**Table 6:** Disease effect on chemokine receptor (CKR) expression in selected neuroinflammatory disorders.

Receptor–ligand axis		Observation	Reference

Receptor^a^	Ligand^b^	CSF immune cell studies	
CCR2	CCL2	The frequency of CCR2+ T cells is increased in adult-onset multiple sclerosis (MS)	(48)
CCR5	CCL5	CCR5+ CCR2+ T cells are selectively enriched in MS only during relapse	(53)
		Increased CCR5 T cell expression in untreated MS	(131)
CCR7	CCL21	In MS, 90% of CSF T cells express CCR7	(80)
CXCR5	CXCL13	Selective accumulation of CXCR5+ memory B cells in CSF, not blood, was found in untreated pOMS	(81)
CXCR3	CXCL10	The percentage of CXCR3+ T cells is not increased in MS or pOMS	(132, 133)
		Increased CXCR3 T cell expression in untreated MS	(131)

*^a^Frequency of CKR-expressing lymphocytes measured by cerebrospinal fluid (CSF) flow cytometry*.

*^b^Increased CSF concentrations*.

### CSF Immunomarkers in CNS Infections

From an immunological standpoint, non-infectious and infectious causes of neuroinflammation are separate, flip side considerations. However, a brief look at intrathecal inflammation in CNS infections affords the opportunity to compare the host inflammatory response to autoantigens vs exogenous microbial antigens (Table 7) (134–159) There were more similarities than differences, given increased CSF concentrations of IL-6, IL-8, and IL-10, but no clear split between bacterial vs viral diseases with the exception of subsclerosing panencephalitis, which stood apart (normal IL-10). The immunological mechanisms of bacterial meningitis (160) and viral meningoencephalitis (161) have been explicated.

**Table 7:** Cerebrospinal fluid chemo/cytokine profile in CNS infections.

Disorder (*n*)	Cytokines						Chemokines				
	IL-				TNF Family		CXCL-			Other ↑	Reference
	6	8	10	17	TNF	BAFF	10	12	13		
**Bacterial**											
pBM (140)	↑		↑		↔						(134)
	↔	↔		↑							(135)
(29)										IL-1β	(136)
								↑		CCL2, CCL4	
										CCL17	(137)

**Bacterial/spirochetal**											
Lyme NB (133)		↑		↑			↑			CCL22	(138)
(21)								↑	↑	IL-18	(139–141)
								↑		CCL2, CCL4	
										CCL7	(137)
pLyme NB (33)									↑		(142)
(20)	↑	↑	↑	↑	↑					IFN-γ, IL-2, CCL2	(143)
(15)		↑		↑			↑			CCL22	(138)
Neurosyphilis (5)									↑		(144)
(47)									↑		(145)

**Bacterial/Mycoplasma with CNS symptoms**											
Mycoplasma (14)	↑	↑								IL-18	(146)

**Viral**											
TBE (36)										IL-1β, MIF, CCL5	(147)
pTBE (37)	↑	↑								INF-γ, IL-4	(148)
Viral E (14)	↑	↑	↔		↔					CCL5	(149)
pViral ME (13)	↑									IL-7, IL-13	(150)
HSV1 (14)	↑	↑	↑			↑	↑			CXCL9, IL-1β, IL-20	(151)
pEV-71 ME	↑									IFN-γ	(152)
pMumps M		↑	↑							IL-12, IL-13, IFN-γ	(153)

HIV/AIDS			↑			↑				CCL3	(154)
pHIV/AIDS					↑					IL-1β	(155)

Rubeola/SSPE (60)			↑		↔		↑			IL-12p40	(156)
(30)	(↓)		↔		↔						(157)
(23)	↑		↔		↔						(158)
										IL-1β	(159)

*Prefix “p,” pediatric; *p/a, mixed pediatric and adult; ↑, increased concentration; ↓, decreased concentration; ↔, normal concentration.

*BM, bacterial meningitis; Lyme NB, neuroborreliosis; Mycoplasma, Mycoplasma pneumoniae; TBE, tick-borne encephalitis; Viral ME, meningoencephalitis; HSV, herpes simplex virus; HIV, human immunodeficiency virus; EV-71 ME, enterovirus-71 meningoencephalitis; Mumps M, mumps meningitis; SSPE, subacute sclerosing panencephalitis; CNS, central nervous system; SSPE, subsclerosing panencephalitis; TNF, tumor necrosis factor*.

#### Bacterial Infections

Cerebrospinal fluid cytokine profiling as a method of discriminating infectious agents and types of meningoencephalitis is gaining traction, especially in diagnostically uncertain cases. The very high concentrations of CSF CXCL13 have been suggested as a diagnostic marker of neuroborreliosis in adults (139) and children (142) and are associated with intrathecal synthesis of borrelial antibodies (162). They are also quite high in neurosyphilis (145), which is also spirochetal. In bacterial meningitis, CSF cytokine profiling may add discriminating value in the differential diagnosis of culture negative cases (134, 163, 164). CSF IFN-γ concentration alone, not 11 other cytokines, was higher in pneumococcal meningitis than meningococcal meningitis (165). In a comparative study of *Streptococcus pneumonia, Neisseria meningitis*, and *Hemophilus influenza*, pneumococcal meningitis was associated with higher CSF IFN-γ and CCL2 concentrations (166).

#### Viral Infections

As to viral encephalitides, increased CSF IL-6, IL-7, and IL-13 may differentiate viral encephalitis from autoimmune NMDAR encephalitis in children (150). CNS Dengue virus and Japanese encephalitis virus infection in children share similar cytokine profiles (167). CSF CXCL8, CXCL9, and CXCL10 are elevated in herpes simplex herpes simplex virus (HSV) 1 encephalitis and HSV2 meningitis, whereas CXCL11 and CCL8 are elevated in HSV2 meningitis alone (151). Among viral CNS meningoencephalitides, enterovirus and lentivirus have comparably higher CSF IL-12 and IFN-γ concentrations, whereas arbovirus is associated with lower CSF cytokine concentrations than other viral etiologies (168). In mumps meningitis in children, the concentrations of CSF CXCL8, IL-10, IL-12, IL-13, and IFN-γ were higher than in other viral meningitides (153). In H1N1 influenza-infected children, only CSF CCL5 was increased in those with encephalopathy compared with uncomplicated influenza (169).

Usually, treatment of the pathogen causes the neuroinflammation to regress, except when a secondary delayed autoimmune process is in play. There is also research on manipulating chemo/cytokines to hasten recovery and improve outcome. The importance of these chemo/cytokines to neurological outcome has been demonstrated experimentally in transgenic knockout animals (170). Animals lacking IL-1β, IL-6, or IL-12 may be resistant to fatal encephalitis or seizures caused by encephalitis, or have reduced neurological severity scores (170).

Human immunodeficiency virus infection (HIV1 and HIV2) is germane here because its predominant route of entry to monocytes and CD4+ cells is *via* the CCR5 co-receptor (171), which can be blocked by “entry inhibitors.” The CCR5-negative phenotype is resistant to HIV infection. HIV1 shifts its tropism from CCR5 to CXCR4 when the disease progresses to AIDS (172). Other HIV strains may use the CXCR4 receptor for entry (172).

The viral story comes back full circle to autoimmunity in that viral infection and viral-induced immunity may initiate autoimmune disease (170) through various mechanisms, such as direct bystander activation, epitope spreading, molecular mimicry, and release of cryptic epitopes (173). NMDAR encephalitis with positive CSF autoantibodies may follow HSV encephalitis several weeks later (174). Whether immunotherapy can prevent the development of autoimmunity following HSVE requires further study (174). The concept of the “infectome”—referring to “…the collection of an individual’s exposures to infectious agents…”—was introduced to trace infectious triggers of autoimmunity and better the understanding of microbial/host interactions in autoimmune diseases (175).

### Applicability of Translating Findings in Adults to the Pediatric Setting

A reasoned discussion should note similarities and differences between patterns in pediatric- and adult-onset neuroinflammatory disorders, but data are limited. In the neuroinflammatory disorders, there were more similarities than differences in the CSF immunomarker pattern: ADEM (agreement on four markers), AE (five markers), NPSLE (six markers). However, the patterns for other markers were not identical. For comparison of CNS infections, the same markers not being measured in various studies. These data emphasize the need to obtain more direct CK/cytokine profiling data from the pediatric populations to allow more comprehensive evidence synthesis.

Pediatric neuroimmunology poses many challenges to that aim which must be surmounted. There are ethical constraints on CSF access—no healthy volunteers. Hence, there are fewer control data and reference ranges. There are technical constraints of smaller CSF volume, although this is not a problem for simultaneous measurement of multiple CSF immunomarkers on small aliquots from CSF samples of 1 ml. Performance of an LP requires sedation in toddlers and young children to prevent contamination of CSF by blood introduced traumatically into the CSF and for compassionate care. More studies of inflammatory mediators have been done in disorders for which LP is a required diagnostic tool (e.g., CNS infections) than in those where it is viewed as optional. The culture of monitoring CSF biomarkers is perhaps less established in pediatric neuroinflammatory disorders. There are pharmacodynamic differences in children based on immune cell development, such as the greater expression of monocytes compared with lymphocytes in infants, which could affect expression of CSF chemo/cytokines. These obstacles, however, can and have been overcome by some researchers in particular disorders. Methodologies and opportunities need to be embraced by the larger pediatric neurology community (176).

## Targeting CKs or Other Cytokines

### Targeting Approaches: Which to Target and How Best to Do It

The ultimate therapeutic goal is to gracefully overcome maladapted, disease-producing biologic processes, induce remission, and re-introduce immune tolerance without toxicity (1). Consistent with the definition of immunotherapy, biological response modifiers treat disease through induction, enhancement, or suppression of an immune response. *Immunomodulation*, defined as modification of an immune system function or induction of immune tolerance through treatment, encompassing immunosuppression or immune potentiation. Various *immunomodulators* may play different roles in the three-pronged targeting of inflammatory mediators: induction, consolidation, and maintenance/post-consolidation.

**Figure 2:**
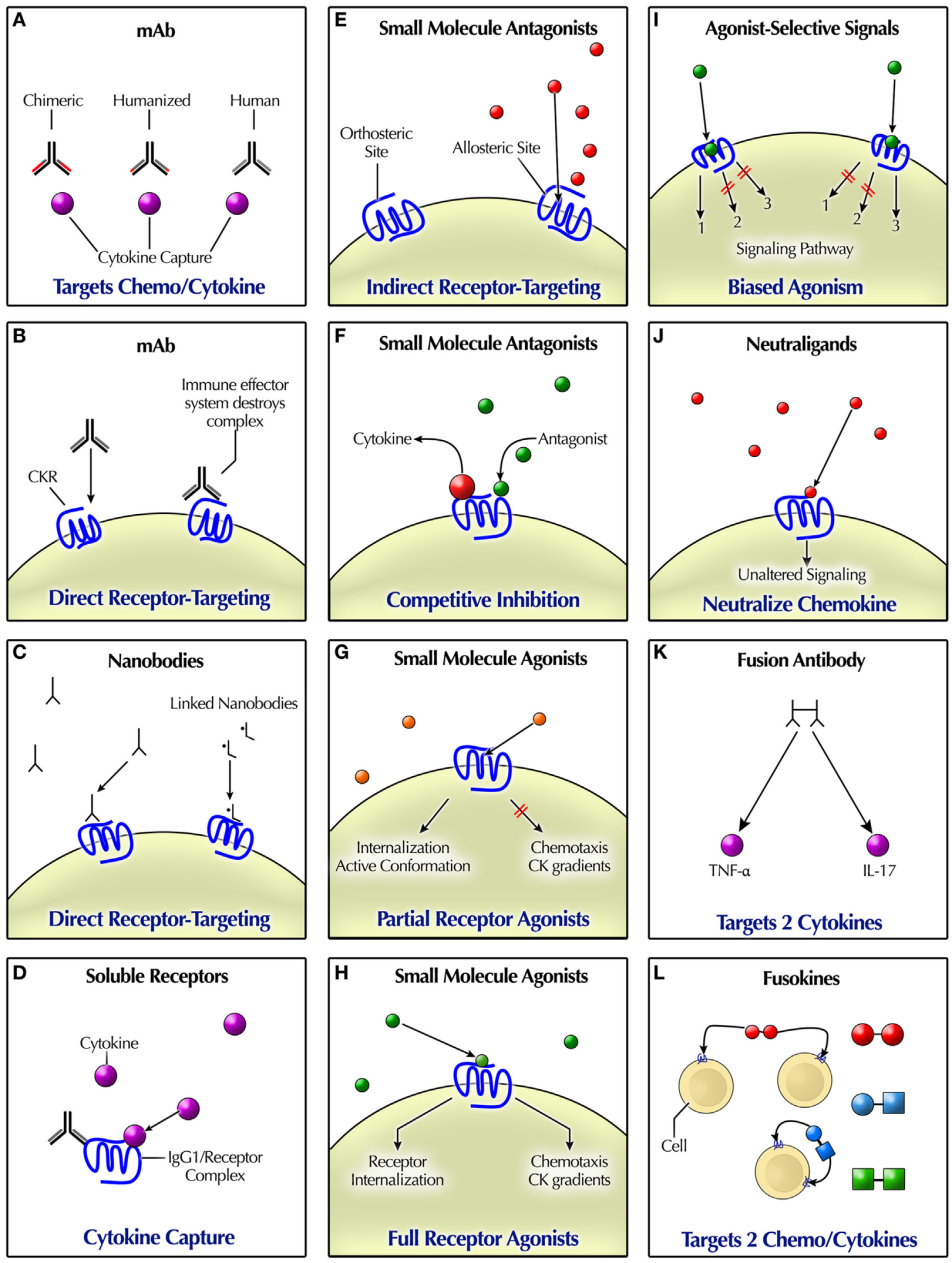
Modalities for pharmacologically targeting chemokine (CK)/cytokine ligands and receptors. **(A)** mAbs can bind to CKs or other cytokines. **(B)** Some mAbs mimic natural ligand binding, acting as agonists or antagonists. The effector immune system removes mAb-targeted cells. **(C)** Nanobodies are engineered “miniature” antibodies. **(D)** Soluble CKR may occur naturally, or they can be engineered as a recombinant fusion protein. Delivery of soluble receptors that bind ligands takes them out of play. **(E)** Indirect receptor targeting or functional antagonism refers to indirect antagonism, such as *via* binding to an allosteric site. By contrast, CKs usually bind at the primary (orthosteric) receptor site, indicative of a syntopic interaction. Some small molecule antagonists bind at an allosteric site. Allosteric agents may possess agonist, antagonist, or neutral effects. **(F)** Competitive inhibition or antagonism occurs when agonist and antagonist binding are mutually exclusive, such as if competing for the same binding site. Non-competitive antagonism refers to the occurrence of agonist and antagonist binding simultaneously. **(G)** Partial agonists have the capacity to produce some but not all of the effects of a full agonist. Their receptor binding may trigger receptor internalization, but perhaps not immune cell chemotaxis or a CK gradient. An inverse agonist reduces the number of receptors in active form. **(H)** A high-efficiency full agonist need only occupy a fraction of total receptors, leaving the rest “spare.” **(I)** Agonist-selective signaling may trigger different signaling based on biased agonism at the receptor. **(J)** Neutraligands bind to the receptor without altering cell signaling. Depicted are small molecules; however, neutral antibodies also exist. **(K)** Fusion antibodies are bispecific, designed to target two differernt cytokines. **(L)** Fusokines either target two cytokines, such as tumor necrosis factor (TNF)-α and IL-17, one cytokine and one CK, or two CKs, such as CXCL10 and XCL1.

From standard-of-care practice, it is already well known that CKs and other cytokines can be immunomodulated by certain powerful “broad-spectrum” biologicals, such as high-dose corticosteroids or corticotropin, which reduce elevated CSF CKs CXCL10, CXCL13 and the cytokine BAFF in pediatric-onset opsoclonus–myoclonus syndrome (OMS) (79–81). “Treat-to-target” strategies hope to avoid the systemic side effects of steroids. It is also known that pathogens manipulate the human immune system, such as encoding for CKs, CKRs, soluble proteins, or extracellular matrix-mimicking components (177). All of these *decoy proteins* prevent CK binding to GAGs and extracellular CK gradients that attract circulating immune cells or CK binding to its cognate receptor (177). The provocative question was posed: if pathogens can co-opt the immune system for their purposes, why can’t we do so for therapeutic reasons? (178). Multiple targeting strategies have been devised, exhibiting a wide spectrum of activity spectrum, inclusive of agonists, partial agonists, and antagonists (Figure 2) (41, 177). Technical details of pharmacologic modulation of CKR function are available elsewhere (172).

#### Monoclonal Antibodies

The clinical mainstay of the chemo/cytokine-targeted approach has been using high-affinity designer *monoclonal antibody* (mAb or moAb) to bind directly to the chemo/cytokine, which resulted in the first mAb clinical usage in 1986 (179) and now more than 500 mAbs. In the trifunctional antibody, the two antigen-binding fragments confer antigen specificity; the single constant fragment, immune effector interaction (180). mAbs were developed in the mouse and then a *chimeric* mouse–human IgG1 (-xi-mab) was created (60–70% human), which combines human Ig constant regions with rodent variable regions. *Humanized* mAbs (>90% human) were followed later by *human* antibodies (100% human), by including the variable Ig regions. Humanized and human mAbs have largely replaced murine and chimeric mAbs for clinical use, and the murine hybridoma technology has been replaced by recombinant DNA technology, phage display, and transgenic mice (180). Therapeutic mAbs (~150 kDa) may directly target the chemo/cytokine or indirectly once its antigen is recognized by anti-dependent cell-mediated or complement-dependent toxicity (173). Most mAbs bind surface receptors without effects of cell signaling or functionality (180). Therapeutic mAbs may be antagonists or allosteric agonists, with binding distinct from the natural ligand (180).

mAbs have been classified by the WHO. The international non-proprietary nomenclature for mAbs can be confusing because the old convention (181) differs from the newer one (182), which is generally shorter. The mAb suffix is “-mab.” Each mAb usually has a unique prefix. Between the two lies a source code (source infix): “-o-mab” for mouse; “-xi-mab” for chimeric; “-lizu-” for humanized; and “-u-mab-” for human. There also may be a target code (target infix): “-limu-” denotes targeting the immune system; “-tu-” indicates tumors; and “-ki-” for IL. As examples, infliximab (infli-xi-mab) would be chimeric, bevacizumab (bevaci-zu-mab) would be humanized; adalimumab (ada-limu-mab) would be human; and canakinumab (cana-ki-umab) would be IL targeting and human, as would ixekizumab (ixe-ki-zumab). The new nomenclature will drop the source infix and devise more specific target inflixes (182).

#### Soluble Receptors

Another approach is to use *soluble receptors* to bind the chemo/cytokine molecule (receptor ligand). This cue is taken from human nature, in which soluble receptors are endogenous and functional. Recombinant fusion proteins combine a key component of the receptor with IgG1 (183). Examples include anakinra (IL-1-Ra) and etanercept (TNF-αR). The recombinant soluble receptor has a much longer half-life than the naturally occurring soluble receptor. Naturally occurring soluble receptors are cleaved from the surface of cells that express the receptor. A new generation of anti-IL-6R mAbs targets both soluble IL-6R (sIL-6R) and membrane-bound (mIL-6R) (184) with improved binding affinity and specificity and reduction in the occurrence of adverse events (185).

#### Small Molecule Antagonists

An additional tactic is use of *small molecule antagonists* (500–600 Da) protein therapeutics, binding proteins, protein antagonists (178). They stabilize the receptor in inactive conformation, usually binding residues in the transmembrane receptor helices, thereby preventing chemo/cytokine binding (42). Alternatively, some small molecule ligands appear not to bind directly to the chemo/cytokine receptor site (non-competitive) but rather to a topographically distinct *allosteric* (allotopic) site (172). Allosteric modulators may be positive (positive allosteric modulators), negative [negative allosteric modulators (NAMS)], or silent/neutral [silent allosteric modulators (SAMS)]. There are numerous patents on low-molecular-weight inhibitors of multiple types of CRs. Anakinra is a recombinant version of an IL-1R antagonist, which works by competitive inhibition of the binding of IL-1 to its receptor.

#### Small Molecule Agonists

Rather than conventional CKR blockade, *small molecule agonists* also are being developed (42). Although they activate the CKR, the functional effect may be partial (partially competitive), not the same as natural receptor ligands, due to different capacities for activating different signaling pathways (42). Allosteric agonists may exhibit *functional selectivity*, such as when the initial post-binding step of receptor internalization is comparable to that of a CK, but the agonist is unable to induce chemotaxis (172).

#### Neutraligands

Chemokine *neutralizing molecules* (“neutraligands” or “neutral ligands”) with anti-inflammatory activity that are not receptor antagonists also are being developed (177). Unlike receptor antagonists, they bind but do not alter CKR basal activity (constitutive activity) (177). Neutraligands mimic pathogen-encoded decoy proteins. There are high-affinity decoy traps for CCL17 and CCL22 (177).

#### Nanobodies (Nbs)/Antibody Fragments

*Nanobodies* were developed to exploit the advantages of mAb miniaturization, avoiding high cost and complexity. These are small “minimalist” antibodies (15 kDa) with a single-domain camelid antibody fragment (186). Nbs have been developed against CCL2, CCL5, CXCR7, CXCL11, and CXCL12 (187). They also can be linked together to form a “biparatopic nanobody.”

#### Bispecific Antibodies (BsAbs)

A developing strategy is more precise targeting of cytokines with *BsAb* (188). The TNF/IL-17 fusion antibody allows simultaneous targeting more than one cytokine (97). It is also possible to inhibit part of a signaling cascade to spare protective cytokine effects, as exemplified by targeting just one of the two TNF receptors (22) or only the trans-signaling of IL-6 triggered cascade (188). A pathogenic cell can be targeted, as in the case of using myeloid specific TNF inhibitor (188). BsAb can also target CKs: a fully humanized IgG-like BsAb for effective dual targeting of CXCR3 and CCR6 has been developed (189).

#### Scaffold Receptor Proteins

Engineered “protein scaffolds” are touted as next-generation antibody therapeutics (190). To eliminate the need for a chimeric intermediate, the complementarity-determining region (“donor”) is inserted into a human antibody scaffold (“acceptor”). The scaffolds are receptor proteins, not immunoglobulins, which are given binding function *via* combinational protein design (190). “Antibody mimetics” are another group of organic compounds (3–20 kDa) that specifically bind antigen but are not related to mAbs. They are based on a naturally occurring protein scaffold template that binds the desired ligand, which is then engineering enhanced (191).

#### Fusokines

*Fusokines*, proteins formed by physical coupling of two cytokines that are functionally different into a single bifunctional polypeptide (192), are in preclinical development. They are purposed for synergistic bioactivity, greater therapeutic potential, and bioavailability than cytokines administered singly or in combinations (193). Fusion of GM-CSF with an IL (IL-2, IL-15, and IL-21), coined “GIFTs,” co-opts the normal signaling machinery of IL receptors, favorably altering the response cell status (194), such as reprogramming B cells into regulatory cells (195). Other fusokines include CXCL10/XCL1 (193) and IL-15/TGF-β (FIST15”) (196). Cytokines also can be fused with CKs (“GMME”), such as GM-CSF with CCL2 or CCL7 (194).

#### Co-Receptor Targeting

**Figure 3:**
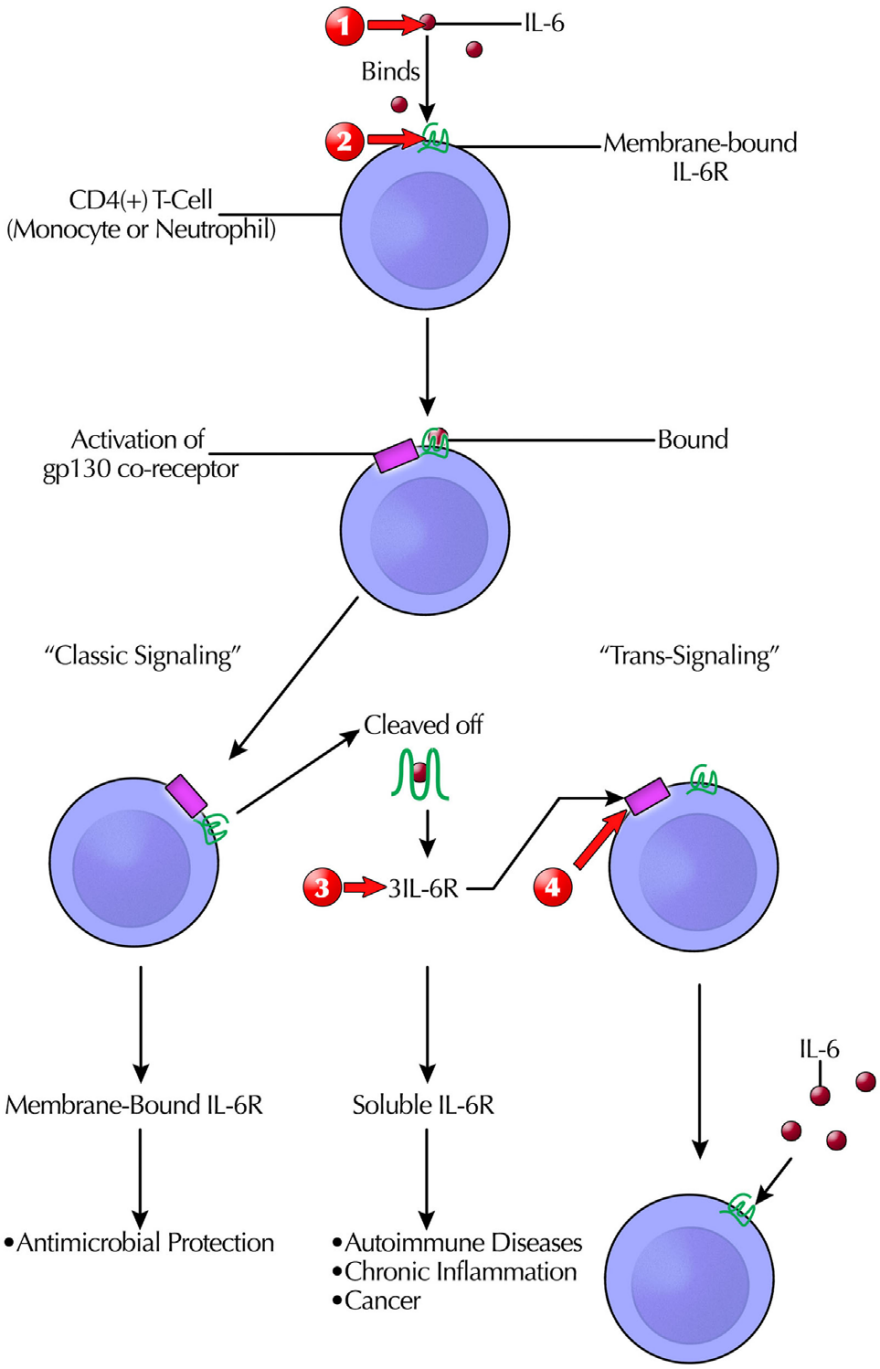
Simplified schema of the complicated targeting IL-6 actions at the receptor binding and signaling/transduction level that give rise to its pleotrophic effects. Only after formation of the three-way complex [IL-6, IL-6R, and glycoprotein 130 (gp130)] is IL-6 signaling initiated. Although gp130 is ubiquitously expressed, fewer cells express IL-6R. Clinically available therapeutic modalities target IL-6 or IL-6R. Another option in clinical trials is blocking sgp130Fc, which does not interfere with host antibacterial defenses. This prevents IL-6 trans-signaling, which is pro-inflammatory. Therapeutic intervention sites are marked with red arrows as follows: (1) anti-IL-6: siltuximab/elselimomab/clazakizumab; (2) anti-mIL-6Rα: tocilizumab/basiliximab/sarilumab; (3) anti-sIL-6R and sIL-6R: sarilumab/tocilizumab; and (4) anti-spg130: sgp130Fc. Abbreviations: STAT, signal transducer and activator of transcription; MAPK, mitogen-activated protein kinase; PI3K, phospitidylinositol-3 kinase; AKT, protein kinase B.

Instead of targeting a CR directly, a new approach is targeting a *co-receptor*, such as receptor-associated gp130, a ubiquitously expressed signal-transducing subunit common to all members of the IL-6 family (185). It is part of the pathway of classic IL-6R signaling. However, naturally occurring sIL-6R may trigger of IL-6 “trans-signaling,” with deleterious effects” (32). Soluble gp130Fc is in phase II trials (32). A simplified schema for mIL-6R and sIL-6R targeting is illustrated (Figure 3) (32, 188, 197).

#### Inflammasome Targeting

Another promising approach is experimental anti-IL-1 targeting of NLRP3 *inflammasome*, a key contributor to the development of neuroinflammation that is amply expressed in the CNS (198). Inflammasomes are three-unit intracellular complexes, constitutive of the innate immune system. CSF NLRP3 is increased after severe TBI in infants and children (197).

#### Transduction Targeting

Agents indirectly affecting cytokines, such as *JAK inhibitors*, are also relevant. These include pan-JAK inhibitors (baricitinib, peficitinib, ruxolitinib, and tofacitinib) and selective JAK inhibitors (decernotinib, filgotinib, and upadacitinib) (199).

#### Other Agents

Serendipity (two-for-one benefit) is not to be discounted. Tacrolimus (FK506), a calcineurin and IL-2 inhibitor, has been found to selectively decrease CSF CXCL10 in an exploratory study of paraneoplastic cerebellar degeneration with anti-Yo and anti-Hu antibodies (99). As CSF CXCL10 is also increased in pOMS and not reduced by conventional agents or steroid sparers (80), the effect may be relevant for OMS and other neurological paraneoplastic syndromes, too.

### Clinical Armamentarium of Immunoneutralizers

Various biopharmaceutical immunomodulators that target CKs or other cytokines have been tried, are in clinical trials, or have been U.S. Food and Drug Administration (FDA)-approved (Table 8) (26, 99, 179, 200–229). Reference to trials in non-neurologic disorders, such as asthma, cancer, Crohn’s disease, HIV, psoriasis, and rheumatoid arthritis (RA), initially may seem off topic, but the treatment of those disorders has fueled trials in neuroinflammatory disorders.

**Table 8:** Overview of some clinical trials/approvals for targeting chemo/cytokines.

Target	mAb/agent	Type	Approval/trial status	Reference
**Cytokines**				
IL-1β	Canakinumab	H	FDA-approved for CAPS (SQ)	(200)
			Phase I/II trial for NOMID	(201)
IL-5	Mepolizumab	Hz	Asthma, COPD	(202)
	Reslizumab	Hz	Asthma, EoE	(203)
IL-6	Siltuximab	C	Multicentric Castleman’s disease	(204, 205)
	Clazakizumab	H	Phase IIb for psoriatic arthritis	(206)
IL-12/23	Ustekinumab	H	Psoriasis; (anti-IL-12, Crohn’s disease); [failed phase II trial for multiple sclerosis (MS)]	(207)
	Briakinumab	H	Psoriasis phase III	(208)
IL-13	Lebrikizumab	Hz	Asthma, s/p phase II (SQ)	(209)
	Tralokinumab	H	Phase III, severe asthma (NCT022813557)	
IL-15	rhIL-15	RH	Advanced solid tumors (SQ)	(210)
IL-17	Secukinumab	H	FDA-approved for psoriasis	(211)
	Ixekizumab	Hz	FDA-approved; PsA (SQ)	(212)
IL-20	NNC0109-0012	H	RA, S/P phase IIa (recombinant)	(213)
IL-22	Fezakinumab	H	Atopic dermatitis (IV)	(214)
IL-23	Guselkumab	H	FDA-approved, psoriasis (SQ)	(215)
TGF-β	Fresolimumab	H	In trials, systemic sclerosis, renal cancer	(216)
TNF	Adalimumab	RH	RA, Crohns (SQ)	(179)
	Certolizumab	Hz	RA phase III	(217)
	Infliximab	C	Neurosarcoidosis (IV)	(218)
	Golimumab	RH	RA, psoriasis (IV) (NCT01362153)	
TNF/IL-17A	COVA322	FP	Phase 1b/2a study in psoriasis (NCT02243787)	(219)
GM-CSF	MOR103	H	MS phase 1b	(220)
BAFF	Belimumab	RH	FDA-approved SLE add-on (failed RA) (SQ)	(221)
			ANCA	(222)
	Blisibimod	FP	SLE, AAV (phase II/III trials)	(223)
BAFF/APRIL	Atacicept	RFP	SLE phase IIb (NCT01972568) (failed RA and MS)	(224)
IFN-α	Rontalizumab	Hz	Subset of SLE, S/P phase II (SQ)	(225)
	Sifalimumab	H	SLE (IV)	(226)
	Arifrolumab	H	SLE phase IIb	(26)

**Chemokines**				
CXCL10	MDX-1100	H	RA, phase II (IV)	(227)
	FK506 (tacrolimus)	Drug	NCT00378326 for PCD (completed)	(99, 228)
	BMS-936557	H	UC, phase II	(229)

*C, chimeric; Hz, humanized (author’s idiosyncratic notation); H, human; FP, fusion protein; RH, recombinant humanized monoclonal antibody; RFP, recombinant fusion protein; CAPS, cryopyrin-associated periodic syndromes; NOMID, neonatal-onset multisystem inflammatory disease (SQ); COPD, chronic obstructive lung disease; EoE, eosinophilic esophagitis; IBD, inflammatory bowel disease; JRA, juvenile rheumatoid arthritis; RA, rheumatoid arthritis; SLE, systemic lupus erythematosus; PsA, psoriatic arthritis; AAV, ANCA-associated vasculitis; UC, ulcerative colitis; IV, intravenous; SQ, subcutaneous; APRIL, a proliferation-induced ligand; FDA, U.S. Food and Drug Administration; PCD, paraneoplastic cerebellar degeneration; TGF, tumor growth factor; TNF, tumor necrosis factor*.

*Source material was gathered using PubMed.gov searches under numerous search terms without restriction to the year of publication, http://Clinicaltrials.gov under disease headings or specific drugs/agents, and various other online resources*.

Systemic anti-cytokine therapy has been daubed the most dramatic therapeutic advance in the modern era of rheumatology (178), although the number of failures exceeded the successes (179). CK blockers have been called “therapeutics in the making” (230), yet the field awaits comparable breakthrough agents.

Although the highly anticipated rituximab therapeutic effect in systemic lupus erythematosus (SLE) was not demonstrated in clinical trials, targeting BAFF was successful (belimumab) (231). IL-1 blocking agents decreased pro-inflammatory cytokines and leukocyte subsets in CSF of pediatric patients with neonatal-onset multisystem inflammatory disease (113), although there were differences in efficacy in the intrathecal compartment (anakinra vs canakinumab). IL-6, a pivotal mediator, has been extensively targeted, both at the level of cytokine and its receptor, with FDA approval in each category. A surrogate marker of anti-IL-6 mAb therapeutic efficacy was inhibition of C-reactive protein (232).

Anti-IFN-γ antibodies have been tested in the treatment of various autoimmune diseases, including MS, schizophrenia, and various autoimmune skin conditions, some with purported clinical benefit (233). However, none have been FDA-approved. Contrariwise, exaggerated type 1 IFN production by DCs has been considered for targeting (60).

Clinical failures or disease exacerbation in some high phase clinical trials have quashed many an immunomarker-guided therapy. The high rate of attrition is multifactorial. Incorrectness of *a priori* hypothesis is one reason. The majority of human clinical trials of treatment for RA with mAbs or synthetic compounds intended to target CK signaling have failed to show clinical efficacy. Of those approved, the clinical response is inadequate in 40–50% (121). One failure was an anti-CCL2 antibody in RA, which paradoxically increased the circulating concentration of CCL2×2,000 (234).

Tumor necrosis factor inhibitors may be associated with CNS demyelinating disorders mimicking MS or myelopathy (235, 236). Serum sickness and encephalitis are other potential complications of anti-cytokine therapy (237). Studies of anti-IL-7; anti-IL-17 RA (receptor; also blocks IL-17 B–F); and anti-CD40-CD40L (toralizumab, H) were pulled due to untoward effects (238). Biologic-induced infections include reactivation of tuberculosis (anti-TNF therapy), viral infections, and reactivation of herpes zoster (26). IFN therapies are immunosuppressive, and *neutropenia* can lead to infections. In patients with SLE, there was an increased frequency of herpes zoster infections in sifalimumab- and anifrolumab-treated patients, and increased influenza rates in anifrolumab-treated patients (239). Tocilizumab can cause intestinal diverticulitis and perforation. Ustekinumab and briakinumab are associated with major cardiovascular events in some patients.

Two clinical trials of rituximab for patients with SLE failed. Although attributed to limitations of trial design and effects of other ongoing treatments, a post-rituximab surge in serum BAFF was found in relapsing patients. Based on the capacity of BAFF to allow autoreactive B cells to survive especially when B cell counts are low, a destructive feedback loop with autoantibody surges has been proposed (28). The authors argue that B cell depletion in SLE should be followed by BAFF inhibition.

### Results in Neurologic Disorders

Although most anti-chemo/cytokine mAbs are not initially developed for CNS application, some are later tested, repurposed, and remarketed. Immunological interrelationships among diseases assist assessment of the probability of cross-utility of the agents.

Tumor necrosis factor neutralization in MS increased relapse frequency and intensity (240). The negative outcome was not predicted by preclinical studies. Although psoriasis and MS share certain immunological features, the IL-12/IL-23 neutralizing mAb ustekinumab was not found effective for MS (207). IL-23 promotes expansion of IL-17 producing Th17 cells. Because ustekinumab targets p40, the subunit common to IL-12 and IL-23, inhibition of IL-12 may also have occurred. IL-12 is an important cytokine for initiation of Th1-mediated inflammatory responses, which is produced by DCs, macrophages, and neutrophils responding to antigen stimulation. Because of the role of IL-17 in neuroinflammatory disorders (241), the anti-IL-17 mAb secukinumab was in a phase II study for MS, but the study was terminated by the sponsor because it had a better anti-17 mAb to test.

The results of targeting of BAFF/APRIL in MS with atacicept were unexpectedly disappointing: it worsened disease activity (NCT00642902, phase II study terminated). In RA, it exerted immunological but not clinical effects. Perhaps the problem is that atacicept impacts regulatory B cells without adequately depleting pathogenic B cells (58).

Adalimumab reduced seizure frequency 10-fold in an open pilot study of 11 patients with RE (242). The improvement was sustained in five patients, three of whom showed no further cognitive decline. RE is otherwise such a devastating, refractory hemiconvulsion/hemiplegia syndrome, it is usually treated by hemispherectomy, but there is limited experience with immunomodulatory treatments early (243).

A pediatric patient with NMO and Sjögren syndrome was successfully treated with tocilizumab (244). In a pilot study of tocilizumab in adults with NMO (245), relapse rate, neuropathic pain, and fatigue were reduced.

In an adult with limbic encephalitis associated with relapsing polychondritis, infliximab treatment was associated with sustained improvement (246). In refractory neurosarcoidosis treated with adjunctive infliximab, most improved clinically but still frequently relapsed (247).

### Targeting CKR/CR

Even a non-exhaustive listing shows more immunomodulatory agents are available with which to target non-CK CRs than CKRs (Table 9) (15, 26, 113, 184, 248–266). Drug discovery initiatives have not delivered on effective agents to selectively manipulate CKs in neuroinflammatory disorders (267). The imputation is that CK binding/signaling is biologically complex and reliant on early synergism between CKs (267). However, CKRs are GPCRs, which have been the most successful target class for drug discovery (268). The concept of CKRs as biomarkers was studied in MS (47).

**Table 9:** Overview of some clinical trials/approvals for targeting chemo/cytokine receptors.

Target	mAb/agent	Type	Approval/trial status	Reference
**Cytokine receptors**				
IL-1Ra	Anakinra	RFP	RA, NOMID, phase II RCT for stroke (SQ)	(113, 248)
IL-2R-α	Daclizumab	Hz	Relapsing multiple sclerosis (MS)	(249, 250)
	Basiliximab	C	Anti-t transplant rejection (IV)	
	Inolimomab	M	Graft-vs-host disease (phase III trial failed)	
IL-4R-α	Dupilumab	H	Uncontrolled asthma (NCT02948959)	
			Atopic dermatitis phase III (SQ)	(251)
IL-5R-α	Benralizumab	Hz	FDA-approved for asthma (SQ)	(252)
IL-6R-α	Tocilizumab	RHz	FDA-approved for RA	(253)
			AE	(254)
			GCA	(255)
			NMO—under study	(256)
	Olokizumab	Hz	RA, phase II	(257)
	Sarilumab	H	FDA-approved for RA (SQ)	(184)
	Saralizumab	Hz	NMO/NMOSD phase II (NCT02073279)	
IL-6R (m & s)	Satrilumab	H	RA; phase II juvenile idiopathic arthritis (NCT027767)	
IL-17R	Brodalumab	H	FDA-approved for psoriasis (SQ)	(258)
Il-31Ra	Nemolizumab	Hz	Eczema (NCT03100344)	
TNFR2	Ethanercept^a^	RFP	RA, JRA, psoriatic arthritis (SQ)	(259)
Type 1 IFN-R	Anifrolumab	H	SLE (phase III trial)	(26)
GM-CSF-Rα	Mavrilimumab	H	RA, phase IIb; phase III	(15, 260)

**Chemokine receptors**				
CCR1	BX471	Drug	Phase II, failed in MS	(261)
CCR4	Mogamulizumab	Hz	T cell lymphoma	(262)
CCR5	PRO 140	H	Phase IIa RCT in HIV	(263)
	Maraviroc	Drug	FDA-approved for HIV infection (not effective in RA)	(264)
CCR9	CCCX282-B	Drug	Phase II, failed in IBD	(265)
CXCR4-α/CXCR7	Plerixafor (AMD3100)	Drug	FDA-approved immunostimulant to mobilize stem cells in lymphoma, multiple myeloma (SQ)	(266)
CXCR4	Ulocuplumab	H	Solid tumors, failed (NCT02472977)	

*M, mouse; C, chimeric; Hz, humanized; H, human; FP, fusion protein; RH, recombinant humanized monoclonal antibody; RFP, recombinant fusion protein; IL-6R (m & s), membrane-bound and soluble IL-6R; IV, intravenous; SQ, subcutaneous; HIV, human immunodeficiency virus; NOMID, neonatal-onset multisystem inflammatory disease (SQ); AE, autoimmune encephalitis; GCA, giant cell arteritis; NMO, neuromyelitis optica; NMOD, neuromyelitis spectrum disorder; JRA, juvenile rheumatoid arthritis; UC, ulcerative colitis; FDA, U.S. Food and Drug Administration; GM-CSF, granulocyte–monocyte colony-stimulating factor; RA, rheumatoid arthritis; SLE, systemic lupus erythematosus; TNF, tumor necrosis factor; IBD, inflammatory bowel disease; Ra, receptor antagonist; α, alpha chain (CD25)*.

*^a^circulating receptor FP, soluble TNFR2 receptor*.

*Plerixator is a partial agonist at CCR4-α and a full agonist of CXCR7*.

*Source material was gathered using PubMed.gov searches under numerous search terms without restriction to the year of publication, http://Clinicaltrials.gov under disease headings or specific drugs/agents, and various other online resources*.

Chemokine receptor-targeted agents may function as agonists, partial agonists, inverse agonists, or antagonists at the receptor site (255). In clinical studies, daclizumab, a humanized mAb *agonist* at the IL-2 receptor (alpha chain) reversed intrathecal increased adaptive immune cells (CD4 and CD8 T cells; B cell) and increased innate lymphoid cells in MS (225). Anakinra, an IL-1 receptor *antagonist*, has recently been applied to the treatment of a few neurological disorders. In a case report of one critically ill child with refractory status epilepticus due to febrile infection-related epilepsy syndrome, anakinra treatment (100). This catastrophic epileptic syndrome is otherwise known for little response to conventional immunomodulatory therapy or antiepileptic drugs (269). Anakinra is also used to treat an autoimmune disorder caused by genetic deficiency of IL-1 receptor antagonist, leaving IL-1 signaling unopposed (270).

The profusion of preclinical studies is exciting, although much of the information is proprietary and unavailable. Anti-CCR1 lacked efficacy in MS (261). CCR2 receptor blockade prevented asthma in a primate model, and anti-CCR3 and anti-CCR4 were tested in asthma (271). Anti-CCR5 and anti-CCR7 have been proposed as future treatments for high-risk CLL (272). In studies of C–C motif CKs, inhibition of CCR6 reduced the severity of experimental autoimmune encephalitis (EAE), an animal model of MS, whether by neutralizing antibodies or a novel receptor antagonist (273). Anti-CCR9 mAbs have been used to demonstrate CCR9 mediation of antiapoptotic signals in cancer. A small molecular inhibitor of CCR9 lacked benefit for inflammatory bowel disease (IBD) (265).

As to C–X–C motif CKs, an mAb to CXCL13 (mAb 5261) was developed (274). Concomitant targeting of CXCL13 and the BAFF receptor mitigated murine Sjögren syndrome (27). Anti-CXCR3 neutralizing antibody may have a role in preventing or treating graft-vs-host disease (275). Targeting CXCR5 in a mouse model of non-Hodgkin lymphoma (276). Anti-CXCR4 (CX549) exhibited anti-inflammatory properties and reduced brain infarction in experimental stroke (277). An engineered, fully human, single-domain antibody-like scaffold or “i-body” has antagonist activity against CXCR4 in humans (278), and there is great interest in targeting CXCR4 (279).

The sIL-23R was delivered in EAE *via* a novel therapeutic strategy: use of adeno-associated virus vectors encoding sIL-23R (280). The anti-IL-23R mAb tildrakizumab also has been developed. The GM-CSF-MCP-1 fusokine selectively inhibited CCR2-expressing lymphomyeloid cells in experimental autoimmune encephalomyelitis (281).

**Figure 4:**
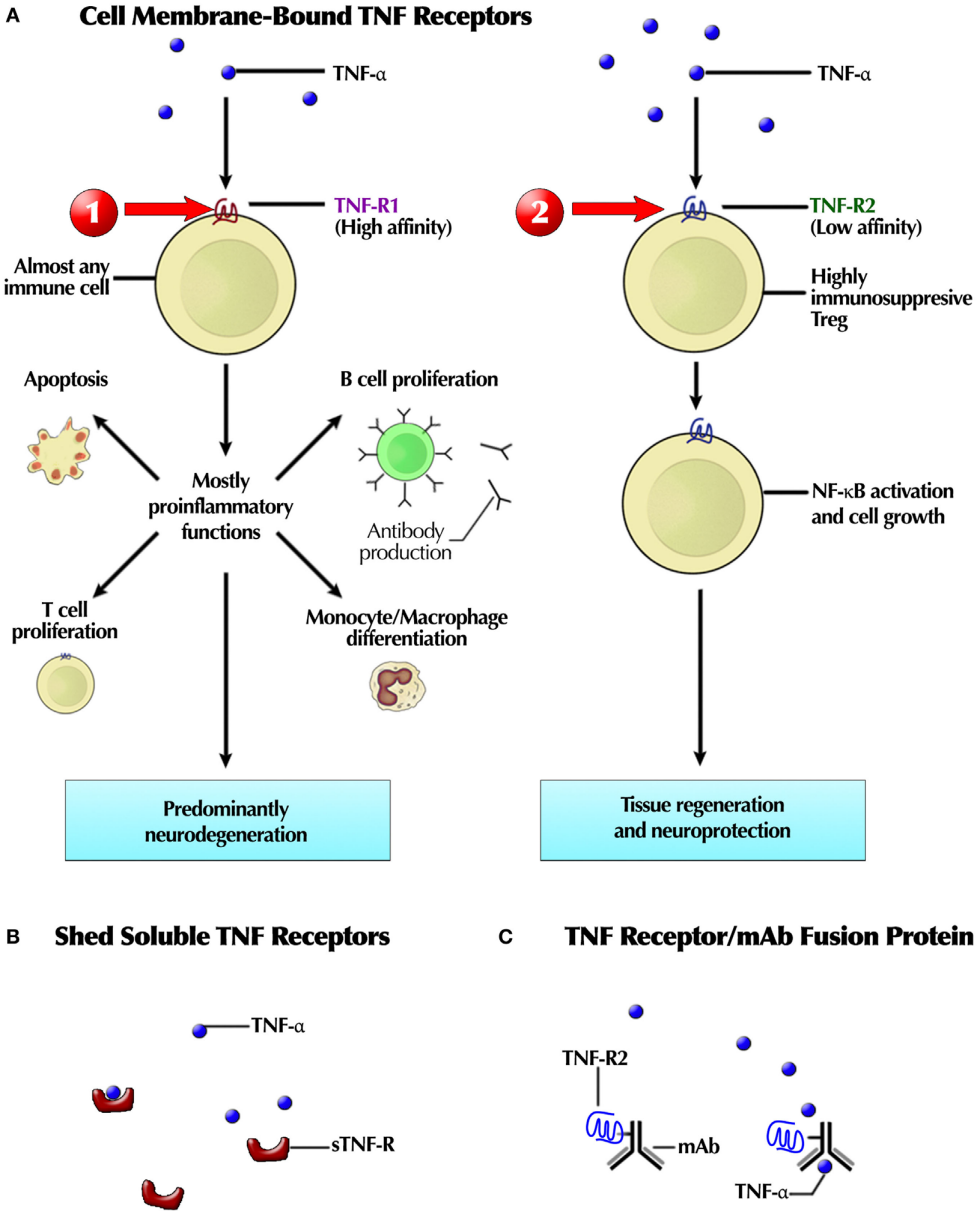
**(A)** Schematic depiction of the targetable differences between TNFR1 and TNFR2 receptor pathways after binding of tumor necrosis factor (TNF), a natural agonist at both receptors. TNF-α, produced by activated macrophages, is a natural ligand at both receptors. TNFR1 receptor stimulation may trigger eventual neurodegeneration, so a selective TNFR1 antagonist may block it. TNFR2 receptor stimulation is associated this tissue regeneration and neuroprotection, hence a selective TNFR2 agonist could also treat neurogenerative diseases. In support of that premise, a selective TNFR2 receptor antagonist has destructive effects: inhibits regulatory T cell (Treg) proliferation, TNFR2 secretion from cells, and promotes T effector cell expansion. **(B)** Endogenous TNF soluble or decoy receptors, shed from membrane-bound TNFR, sequester TNF-α, preventing its inflammatory effects. **(C)** A receptor-Fc construct, combining a TNFR2 fragment with an mAb. This recombinant fusion protein is ethanercept. Red arrows indicate therapeutic intervention sites: (1) TNFR1 antagonist and (2) selective TNFR2 agonist. Not shown is the therapeutic potential of a TNFR2 antagonist for the treatment of cancer, inhibiting Treg proliferation, soluble TNFR2 secretion from normal cells, but increased effector T cell expansion, as demonstrated *in vitro*. Thus, the target shifts with the therapeutic purpose and disease.

Because TNF-α binds both to TNFR1 and TNFR2, targeting TNF-α itself would be expected to have different downstream effects than selectively targeting either receptor (Figure 4). Such factors contribute to the complexity of choosing the best target. *In vitro*, an agonist mAb enhanced TNFR2 signaling and rescued human neurons from oxidative stress-induced cell death (282). Conversely, TNFR2 antagonists inhibit regulatory T cell (Treg) proliferation, which has application in cancer therapy (283).

## Cytokine Delivery as Immunotherapy

Although targeting chemo/cytokines has been the focus of the discussion thus far, direct delivery of cytokines is another therapeutic strategy. The most clinically and commercially successful venture has been with type 1 IFNs, a subclass of cytokines. IFN-α-1a and IFN-α-1b, as well as IFN-β-1a and IFN-α-2b are licensed and well-known therapies for MS (284). Abnormal upregulation of type 1 IFN signaling and gene expression stimulated by IFN have been tied to “interferonopathies,” a common moniker for a group of CNS diseases that includes AGS (284), a pediatric-onset epileptic encephalopathy characterized by increased CSF IFN-α, CCL2, and CXCL10 (104). Anti-type 1 IFN therapy has been attempted in SLE (238).

Interferon-gamma, the sole type 2 IFN, binds to the IFN-γ-R1 and IFN-γ-R2 receptors (also denoted IFNGR1 and R2). IFN-γ-1b is FDA-approved for chronic granulomatous diseases and osteopetrosis (285). A phase III study (NCT024155127) of IFN-γ-1b for the treatment of Friedreich ataxia has been completed recently, but without study results yet, based on positive results from as phase II study (143). Treatment with IFN-γ exacerbated MS (286).

There are fewer non-IFN cytokine therapies. Low-dose IL-2 was used as a treatment for refractory autoimmune encephalitis in 10 adults, based on the assumption that low-dose restores the balance of regulatory and effector T cells, with clinical improvement in 6 (287). In preclinical studies, administration of G-CSF in a murine model of Friedreich ataxia resulted in clinical improvement and reduction in inflammation and gliosis (288). IL-10 is such a powerful counteractant of pro-inflammatory cytokines, one would hope there is more progress in this area.

Although they would provoke, not treat, neuroinflammation, several cytokines are used to treat cancer. They include high-dose IL-2, IFN-γ, IL-15, and GM-CSF (289). By contrast, IL-1, TNF-α, IL-4, and IL-6 were ineffective.

Another concept is administering the cytokine in combination with an inhibitor of its breakdown. Combination of IFN-β with inhibitors of proteinases that degrade IFN-β has been conceived for MS (290). Such approaches may be applicable with other cytokines.

## Considerations in Designing Future Clinical Trials for Neuroimmunologic Application

### Avoiding Targeted Treatment Failure

Some of the more common reasons for failure of targeted treatment have been explicated (291). Several result from miscalculation of pharmacodynamic effects. Unsound target selection is common (291). Because biologic targets are dynamic, not static, therapeutic approaches must be highly anticipatory of downstream effects, particularly negative ones. Undesirable “off-target effects” can be stimulated (177) due to low specificity or rapid clearance rates of the ligand/drug (255). Insufficient dosing to achieve therapeutic receptor saturation can result in false negative results (291). Redundant or opposing chemo/cytokine functions may negate the therapeutic effect: receptor antagonists tend to have some degree of undesirable partial or inverse agonistic activities (177). Target validation in experimental animals may be problematic because pertinent human chemokine receptor–ligand pairs may have different roles (291).

Impractical clinical trial decisions are another reason for avoidable treatment failure. Changing successful clinical trial endpoints from a phase II trial to different ones in a phase III trial may compromise the latter (291). Sometimes a pharmacokinetic study would have been advisable. The clinical trial conditions may not be comparable or translatable to the “real world” clinical setting. Finding both active and passive controls can be challenging. Post-marketing adverse events are often unpredictable.

### Strategic Insights

Clinical and preclinical studies of chemo/cytokine-targeted therapy have provided strategic insights for the next level of therapeutic intervention, which over time will become more nuanced (Table 10) (27, 58, 177–179, 187, 188, 206, 214, 237, 268, 285, 292). The fluctuating nature of responses to immunotherapy, the unintended consequences, and natural remission are cautionary sign posts along the road to innovation. There are also differences in biologicals and synthetics to consider.

**Table 10:** Strategic insights from clinical trials and preclinical studies of chemo/cytokine-targeted therapy.

Many neuroinflammatory disorders share particular CSF immunobiomarkers, so development of new drug/mAb for markers stands to benefit more than one disorder (178, 292) Concomitant neutralization of chemokine (CK) bioactivity and chemokine receptor (CKR) blockade may be more effective than neutralization of CK alone (27) Simultaneously blocking pro-inflammatory actions of CKR and activating anti-inflammatory actions (187) Some immunomarker-targeted therapies perform best as adjunctive to standard-of-care immunotherapies (206, 214) As neuroinflammatory disorders involve multiple inflammatory mechanisms, targeting a single immunomarker or pathway may be insufficient (292) Targeting a cytokine/CK may have a different clinical effect than targeting the receptor (285) Clinical trials of some promising neutralizers have been curtailed due to lack of sufficient efficacy, unexpected increase in neuroinflammation, side effects, or remarketing strategies (58) High levels of receptor occupancy by the blocking agents may be required to prevent signaling (179) Chemo/cytokine functions can overlap, so targeting one may yield an incomplete effect (179) Targeting CKRs on T cells would have different consequences for regulatory T cells (may be counterproductive) than for effector T cells (may be therapeutic) (268) More specific cytokine inhibitors, sparing protective immunoregulatory function, may result in fewer unwanted effects than cytokine ablation (188) Adverse events may result from either on-target or off-target treatment effects (177) Stand-alone agents targeting individual cytokines are less likely to work than combination targeting (188) The more disease-specific the treatment, the less non-specific adverse events (237)

Under the “approach cautiously” category fall the pleotrophic cytokines (IL-2, IL-6, IL-10, IL-12, IL-13, IL-21, and IL-25). They are multipotent and multipurposed. There may be unwanted consequences of ablating or globally blockading these cytokines, prompting the development of more specific inhibitors to preserve protective immunoregulatory functions (188).

It would seem logical to apply agents to those disorders in which the specific CSF biomarker elevation has been demonstrated rather to ones in which the concentration is normal. Elevation of chemo/cytokine concentrations by themselves, of course, do not prove pathogenicity, and all biomarkers are not measured in all disorders, so data are sometimes scant or lacking. G-CSF was increased in NMO, ADEM, and NPSLE; GM-CSF in NPSLE, stroke, and SCA. APRIL was elevated in NPSLE, MOG(+) MS, and NMO (not pOMS). IL-13 was increased in MS, NMO, SCA, MSA-C, and AGS; IFN-γ in ADEM and AE.

The relation of BAFF and APRIL in neuroinflammatory disorders is curious: they often do not sort together. In the case of NPSLE, APRIL is elevated, BAFF less so, and BAFF is increased in plasma (29). In pOMS, CSF BAFF and CSF/serum BAFF ratio are elevated, but APRIL is not (79, 94). Responses to therapeutics differ as well: IVIg increased serum APRIL, not BAFF, in pOMS (79). These are important considerations in deciding in which disorders belimumab may be useful.

Ligand–receptor dynamics, although complex, can be further exploited. The systemic concentration of soluble IL-6 receptors (sIL-6R) normally exceeds that of IL-6 (188). Most harmful effects of IL-6 appear to be mediated by sIL-6R, whereas the host-protective functions of IL-6 are mediated by membrane-bound IL-6R (293). It would be informative and useful to start measuring concentrations of pertinent, naturally occurring, soluble chemo/cytokine receptor concentrations (30–32). As therapies targeting these soluble receptors advance, patient selection would benefit. The clinical availability of reference laboratories and costs would require evaluation.

Many existing drugs can be repurposed to treat neuroinflammation. The anti-HIV drug maraviroc, which prevents HIV entry into the cell through CCR5, has been proposed to blockade CCR5 in neuroinflammatory diseases (264). It is an available and well-tolerated approved drug. It may become feasible to screen for CCR5 receptor polymorphisms.

When CXCL13/CXCR5 targeting is available to neuroimmunology researchers, its use has been suggested for MS (83), NMDAR encephalitis (294), and OMS (81). Understanding the alternative roles of CXCR3 signaling will be required, however. In glial cells, CXCR3 improves EAE by reducing CNS-infiltrating Th17 cells through prevention of a pro-Th17 cytokine milieu (295). Also, CCL2 and CCR2 have been proposed as selective targets for the treatment of MS (296).

Leaving aside varied pharmacokinetic factors associated the wide spectrum of patient ages in neuroinflammatory disorders, there are several important pharmacodynamic considerations. What is the accessibility of the target? Activated intrathecal B and T cells become embedded in brain tissue in progressive MS, but not RR-MS, where they may be more difficult to rout. Early treatment prior to that stage may have better clinical impact. What is the tolerability of chronic pharmacologic blockade? Possible compensatory mechanisms should be anticipated. What are the specific windows of therapeutic opportunity, such: timing, context, developmental stage? What are the appropriate response-predictive biomarkers? There is a growing need for proof-of-concept studies, especially in orphan diseases for which there is no animal model.

Monitoring for anti-mAb and anti-drug antibodies, such as human antihuman or HAHA, spawned by their immunogenicity is an important concern for effective treatment, especially when there is a reduction in efficacy or loss of response (237). The percent of treated patients with antidrug antibodies is highly variable. In the case of IBD, infliximab ranked highest in antibody frequency (65%), but the antibodies were also detected after treatment with adalimumab, certolizumab pegol, golimumab, and ustekinumab (237).

### Navigating Constraints in Pediatric Drug Trials

Clinical trials in pediatric-onset neuroinflammatory disorders require special handling. The pediatric population is designated as “vulnerable,” resulting in restrictions on clinical research. Ethical and federal regulatory issues surround vulnerable populations in pragmatic clinical trials (297). As to immunotherapy, there is a reliance on drugs tested in adults, especially FDA-approved drugs for other clinical indications, and perhaps fewer assessable immunopharmacological agents for clinical trials and use in pediatric neuroinflammatory disorders. Also, the propensity for pharmacokinetic differences between children and adults is well known. Pediatric medication safety research faces the difficulty of assessing child-reported adverse drug events (298).

### *In Vivo* Imaging of Human Neuroinflammation

In the foreseeable future, molecular imaging with radiolabeled ligands could facilitate patient selection for targeted therapy. Radiolabeled CXCR4 ligands have been developed recently (279). In experimental animals, inflammasome activation has been revealed by *in vivo* imaging (299). ^99m^TNFR2-Fc-IL-1ra was developed for use with single-photon emission computed tomography imaging to image inflammation *via* TNF and IL-1 pathways in mice (300). Neuroimaging methods can target the activation of immune cells in the CNS with TSPO tracer [(11)C]PB28 (301). Iron oxide nanoparticles and MRI can be used to track CNS infiltration by monocytes and other circulating immune cells (301).

## Future Perspectives

Targeting dysregulated CKs and other cytokines or their receptors is a provocative and growing therapeutic line of approach with complex implications, and an emerging era for pediatric neuroinflammatory disorders. The goal is to apply gathering evidence to treatment optimization with continual contemplation of potential consequences of targeted engagement. Central to this process is developing algorithms using biomarker criteria and a focused set of molecular measures. Several trends are driving the emergence of personalized medicine, for which biomarkers play a pivotal role. Greater understanding of disease mechanisms and pharmacodynamics is essential, and collecting data must be embraced by treating physicians now that standard tests for defining neuroinflammation are now obsolete. Tailored treatment approaches can be based on clinical and biological factors revealed by immunobiomarker interrogation of neuroinflammatory diseases.

Historically, targeted therapies being tested for neuroinflammatory disorders were developed for other disease applications and repositioned. Some have provided clinical benefit; others merely show proof-of-concept to date; some failed. Taken together, the present observations support the further application of agents developed for other disorders in clinical trials for neuroinflammation disorders given the redoubtable onslaught of disease. Because many inhibitor projects fail, it is crucial to obtain as much information about the target and its role in the disease immunopathophysiology before starting a drug-development program.

Cerebrospinal fluid biomarker studies thus far have revealed several lessons: patients with extreme levels of CSF biomarkers may show higher morbidity or mortality, CSF multi-analyte profiling may be more clinically helpful than single markers, and the use of several may have the best predictive value. More success has been obtained when non-CK cytokines and/or their receptors are targeted, and there have been no CK-targeting drugs licensed for treating neuroinflammation. FDA approvals often specify for individuals with previous inadequate response to two or more DMTs or failure of standard immunotherapy, so early intervention has not been adequately tested. More development of “risk-based” treatment approaches, used successfully in cancer therapeutics, is needed for neuroinflammatory disorders.

In most cases, CSF CK/cytokine data in pediatric-onset neuroinflammatory disorders are lacking, preventing systematic comparison with their adult-onset counterparts. Herein is a call-to-action to gather systematic data on CSF cytokine and CK concentrations particularly in pediatric-onset MS, neuropsychiatric lupus, and NMO. Literature on how to design phase II clinical trial designs for initial biomarker validation is already available (302). CSF CK/cytokine biomarkers should be added to clinical trials of new agents for neuroinflammation (9). Among more recent concepts, treating neuroinflammation alone may be insufficient, and neuroprotection of the innate immune system may be necessary (303). The goal of successfully treating serious diseases once considered as “undruggable” should propel research into the future of biomarker-targeted immunotherapeutics, optimistically renamed “difficult-to-drug” or “yet-to-be-drugged” (304).

## Author Contributions

The author performed the literature searches and wrote the manuscript.

## Conflict of Interest Statement

The author declares that the research was conducted in the absence of any commercial or financial relationships that could be construed as a potential conflict of interest. The reviewer KK and handling editor declared their shared affiliation.

## Supplementary Material

Figure S1 Cytokine concentrations in **(A)** cerebrospinal fluid (CSF) and **(B)** serum. Means with standard deviation (SD) in parentheses (*larger figure*) and medians with interquartile range in parentheses (*insert*) are ranked. Other medians could not be calculated due to the number of samples with undetectable levels.

Figure S2 Cytokine detection rates in CSF and serum in the entire dataset. Percent detectability was defined as the percentage of samples in which a given cytokine could be detected. The order of the cytokines displayed is from the highest to the lowest CSF detection rate.

## Abbreviations

AAV, ANCA-associated vasculitis; ADEM, acute disseminated encephalomyelitis; AE, autoimmune encephalitis; AGS, Aicardi–Goutières syndrome; AIDP, acute demyelinating polyradiculoneuropathy; ALD, adrenoleukodystrophy; APRIL, a proliferation-induced ligand; ACKR, atypical chemokine receptor; AKT, protein kinase B; BAFF-R, B cell-activating factor receptor; BCMA, B cell maturation antigen; Breg, regulatory B cell; BsAb, bispecific antibody; C, chimeric; CAPS, cryopyrin-associated periodic syndromes; CCL, C–C cryopyrin-associated periodic syndromes; chemokine ligand motif, C-CSF, granulocyte colony-stimulating factor; CDR, complementarity-determining region; CIDP, chronic inflammatory demyelinating polyneuropathy; CIS, clinically isolated syndrome; CK, chemokine; CKR, chemokine receptor; CNS, central nervous system; CSF, cerebrospinal fluid; chemokine, chemoattractive cytokine; CXCL, C–X–C chemokine ligand motif; CX3CL1, C–X–3C chemokine ligand motif; DC, dendritic cell; EV-71, enterovirus-71; FDA, U.S. Food and Drug Administration; Fab, antigen-binding fragment; FC, single constant fragment; FIRES, febrile infection-related epilepsy syndrome; FP, fusion protein; GABA, gamma-aminobutyric acid; GAGs, glycosaminoglycans; GBS, Guillain–Barré syndrome; gp130, glycoprotein 130; GPCRS, G-protein-coupled receptors; GM-CSF, granulocyte–monocyte colony-stimulating factor; H, human/humanized; HIV, human immunodeficiency virus; IEM, inborn error of metabolism; IFN, interferon; IL, interleukin; IFN-γ, interferon-gamma; IL-Ra, IL-receptor antagonist; IS, infantile spasms; IV, intravenous; IVIg, intravenous immunoglobulin; JAK, Janus kinase; JRA, juvenile rheumatoid arthritis; LP, lumbar puncture; Lyme NB, neuroborreliosis; MAPK, mitogen-activated protein kinase; mAb, monoclonal antibody; MLD, metachromatic leukodystrophy; MOG, myelin oligodendrocyte glycoprotein; MS, multiple sclerosis; MSA-C, multiple system atrophy cerebellar type; NAMS, negative allosteric modulators; Nb, nanobody; NF-κB, nuclear factor kappa-light-chain-enhancer of activated B cells; NK, natural killer; NKT, natural killer-like T cell; NMDAR, *N*-methyl-d-aspartate receptor; N-Beçhet, neuro-Beçhet disease; NMO, neuromyelitis optica; NOMID, neonatal-onset multisystem inflammatory disease; NPSLE, neuropsychiatric systemic lupus erythematosus; OMS, opsoclonus–myoclonus syndrome; PAMS, positive allosteric modulators; PCD, paraneoplastic cerebellar degeneration; PI3K, phosphatidylinositol-3 kinase; pPPH, post-hemorrhagic hydrocephalus; RA, rheumatoid arthritis; RE, Rasmussen encephalitis; RFP, recombinant fusion protein; RH, recombinant humanized monoclonal antibody; RR-MS, relapsing–remitting multiple sclerosis; SAMS, silent allosteric modulators; SC, Sydenham chorea; SCA, spinocerebellar atrophy; Sjögren syndrome-associated meningoencephalitis; SLE, systemic lupus erythematosus; SP-MS, secondary-progressive MS; SSPE, subsclerosing panencephalitis; SQ, subcutaneous; STAT, signal transducer and activator of transcription; TACI, transmembrane activator and calcium modulator and cyclophilin ligand interactor; TBE, tick-borne encephalitis; TBI, traumatic brain injury; TGF, tumor growth factor; Th, T helper cell; Thf, T follicular helper cell; TNF, tumor necrosis factor; Treg, regulatory T cell; UC, ulcerative colitis; viral ME, meningoencephalitis.
