# Supplementary material for: Advances in Biomarker-Guided Therapy for Pediatric- and Adult-Onset Neuroinflammatory Disorders: Targeting Chemokines/Cytokines
Source: Front Immunol. 2018 Apr 4;9:557. doi: 10.3389/fimmu.2018.00557 (PMC5893838; doi:10.3389/fimmu.2018.00557)
Supplement: Supplementary file 1 [file table_1.PDF]

TABLE 1. Chemokine nomenclature.

Structural name	Common/historical name	
**C – C motif**		
CCL1	I-309	
CCL2	MCP-1	Monocyte chemoattractant protein-1
CCL3	MIP-1α	Macrophage inflammatory protein-1α
CCL4	MIP-1β	Macrophage inflammatory protein-1β
CCL5	RANTES	Regulated on activation normally T cell expressed and secreted
CCL6	HCC-4	
CCL7	MCP-3	Monocyte chemoattractant protein-3
CCL8	MCP-2	Monocyte chemoattractant protein-2
CCL9	(murine)	
CCL10		
CCL11	Eotaxin-1	Eosinophil chemotactic protein-1
CCL12	MCP-5	Monocyte chemoattractant protein-5
CCL13	MCP-4	Monocyte chemoattractant protein-4
CCL14	HCC-1	
CCL15	MIP-5	Macrophage inflammatory protein-5
CCL16	MTN-1	Monotactin-1
CCL17	TARC	Thymus and activation regulated chemokine
CCL18	MIP-4	Macrophage inflammatory protein-4
CCL19	MIP-3β	Macrophage inflammatory protein-3β
CCL20	MIP-3α	Macrophage inflammatory protein-3α
CCL21	SLC	Secondary lymphoid tissue derived cytokine
CCL22	MDC	Macrophage-derived chemokine
CCL23	MIP-3	Macrophage inflammatory protein-3
CCL24	Eotaxin-2	Eosinophil chemotactic protein-2
CCL25	TECK	Thymus expressed chemokine
CCL26	Eotaxin 3	Eosinophil chemotactic protein-3
CCL27	CTACK	Cutaneous T cell-attracting chemokine
CCL28	MEC	Mucosae-associated epithelial chemokine
**C- X - C motif**		
CXCL1	GRO-α	Growth-related oncogene
CXCL2	GRO-β	Growth-related oncogene
CXCL3	GRO-γ	Growth-related oncogene
CXCL4	CD184	(Platelet-derived chemokine)
CXCL5	(murine)	
CXCL6	GCP2	Granulocyte chemotactic protein 2
CXCL7	TCK-1	Thymus chemokine-1
CXCL8	IL-8	Interleukin-8

CXCL9	Mig	Macrophage inflammatory protein-1γ
CXCL10	IP-10	Interferon-γ-inducible protein 10
CXCL11	I-TAC	Interferon inducible T cell alpha chemokine
CXCL12	SDF-1α	Stromal cell-derived factor-1alpha
CXCL13	BCA-1	B cell attractant-1
CXCL14	MIP-2γ	Macrophage inflammatory protein-2γ
CXCL15	Lungkine	
CXCL16	ROCK1	Rho associated coiled-coil containing protein kinase-1
CXCL17	GPR35	G protein-coupled receptor ligand 35
**C- motif**		
XCL	Lymphotactin	
**C - X - 3C motif**		
CX3CL1	Fractalkine	
