# Supplementary material for: Advances in Biomarker-Guided Therapy for Pediatric- and Adult-Onset Neuroinflammatory Disorders: Targeting Chemokines/Cytokines
Source: Front Immunol. 2018 Apr 4;9:557. doi: 10.3389/fimmu.2018.00557 (PMC5893838; doi:10.3389/fimmu.2018.00557)
Supplement: Supplementary file 2 [file table_2.PDF]

SUPPLEMENTARY TABLE 2. Listing and cameos of pediatric-onset neuroinflammatory disorders cited in the review.

Disorder	Brief description
Aicardi-Goutières Syndrome	Rare genetic disorder of pediatric-onset: epileptic encephalopathy, spasticity, cognitive impairment. Chronic CSF inflammation
ALD	Adrenoleukodystrophy: progressive CNS demyelination with adrenocortical failure. Cerebral form most common
Anti-Hu/Anti-ANNA1	Debilitating seizures, cognitive impairment, behavioral problems. Associated with neuroblastoma in some children, paraneoplastic. May or may not be immunotherapy-responsive.
FIRES	Catastrophic febrile infection-related epilepsy syndrome. Immunomodulation treatment sometimes helpful. Onset ages 3 – 15 years
Infantile Spasms	Devastating epilepsy syndrome, onset between 4 - 7 months. Distinctive clinical "spasms," chaotic EEG hypsarthythmia. Identifiable cause in 75%. Often steroid- or ACTH-responsive but high risk of cognitive impairment
Krabbe disease	Globoid cell leukodystrophy due to galactosylceramide β-galactosidase deficiency. Developmental regression, progressive hypertonicity, hyporeflexia, seizures, and blindness.
Miller Fisher	Acute syndrome of ataxia, areflexia, opthalmoplegia, demyelination. Campylobacter jejuni serotype 0:19. IVIg- or plasma exchange-responsive
MLD	Metachromatic leukodystrophy from arylsufatase A enzyme deficiency (sulfatide lipidosis). Most infantile or juvenile onset, but some adults. Progressive central and peripheral neurologic signs
NOMID	Neonatal-onset multisystem inflammatory disease. Multiple inflammatory markers.

NORSE	New onset refractory status epilepticus. Prolonged status epilepticus in children or adults
OMS	Opsoclonus, myoclonus, ataxia with hallmark irritability. Usual onset in toddlers. Associated with neuroblastoma in 50% of cases. Usually steroid- or ACTH-responsive
Ophelia Syndrome	Paraneoplastic syndrome linked to Hodgkin lymphoma and mGluR5 antibodies (not specific). May onset in teens
Rasmussen Encephalitis	Refractory inflammatory encephalopathy with hemiconvulsions and hemiplegia. Standard immunotherapy rarely successful
ROHHAD	Rapid-onset obesity, hypothalamic dysfunction, hypoventilation (potentially fatal), autonomic dysregulation. Sometimes associated with neural crest tumor and paraneoplastic. Positive CSF oligoclonal bands and responses to immunotherapy.
SREAT	Steroid-responsive encephalopathy with autoimmune thyroiditis. May present with convulsions, confusion, hallucinations, gait and memory impairment, or coma in teens.
SSPE	Chronic measles encephalitis: seizures, EEG-periodic spike-wave bursts associated with myoclonic jerks; spasticity, seizures. Elevated antibody titer to rubeola in CSF. Treated with intraventricular α-interferon
Sydenham Chorea	A major criterion for acute rheumatic fever. Most common acquired cause of chorea in children, resulting from Group A, β-hemolytic streptococcal infection. Chorea, hypotonia, emotional lability. Antibodies to basal ganglia or dopamine receptor 2
