# Supplementary figures and images for: Advances in Biomarker-Guided Therapy for Pediatric- and Adult-Onset Neuroinflammatory Disorders: Targeting Chemokines/Cytokines
Source: Front Immunol. 2018 Apr 4;9:557. doi: 10.3389/fimmu.2018.00557 (PMC5893838; doi:10.3389/fimmu.2018.00557)

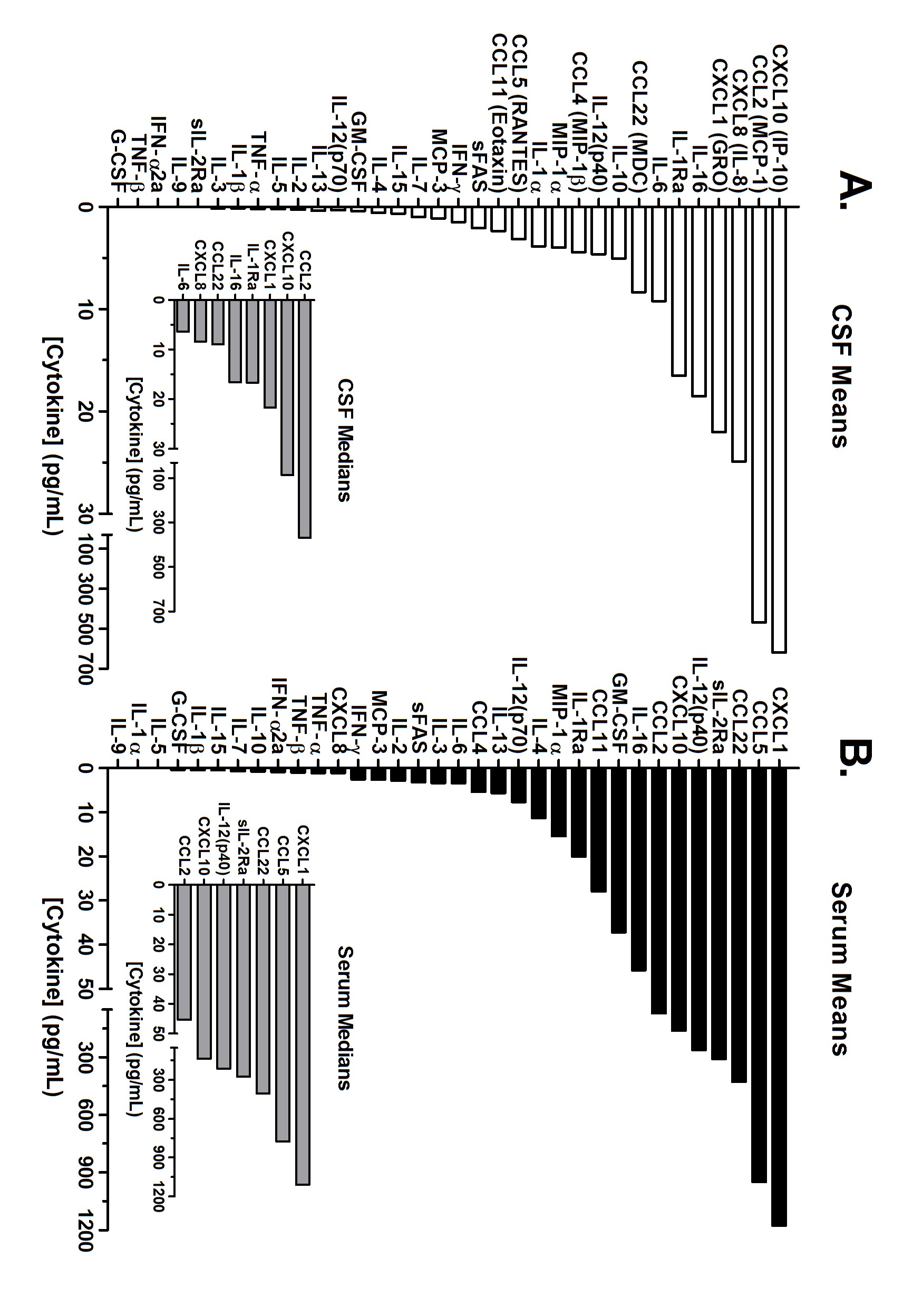

Supplement: Figure S1 — Cytokine concentrations in (A) cerebrospinal fluid (CSF) and (B) serum. Means with standard deviation (SD) in parentheses (larger figure) and medians with interquartile range in parentheses (insert) are ranked. Other medians could not be calculated due to the number of samples with undetectable levels. [file image_1.jpeg]

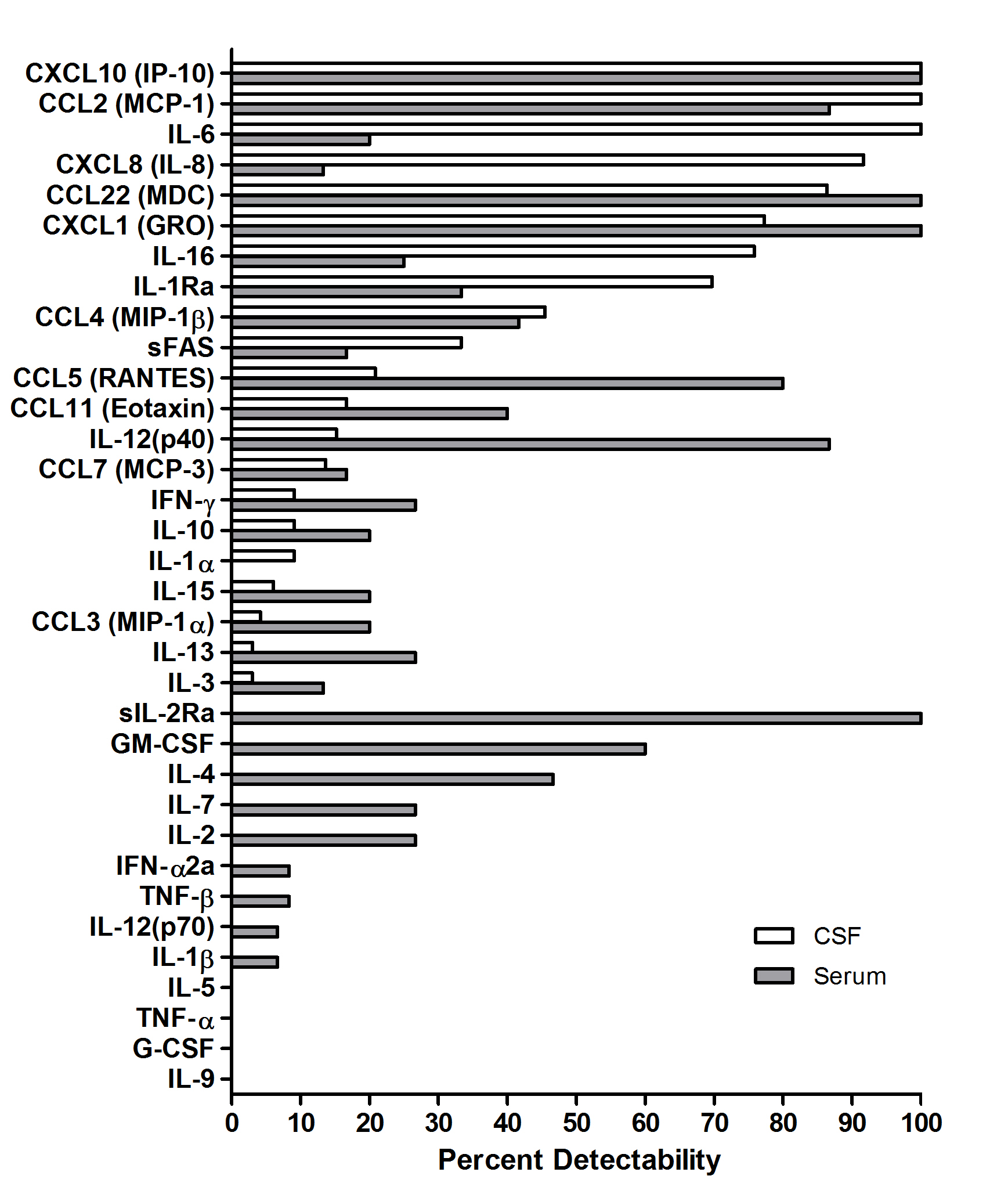

Supplement: Figure S2 — Cytokine detection rates in CSF and serum in the entire dataset. Percent detectability was defined as the percentage of samples in which a given cytokine could be detected. The order of the cytokines displayed is from the highest to the lowest CSF detection rate. [file image_2.jpeg]
